# MethyAnno: An Interpretable Automated Annotation Method Leveraging Multi‐Scale Information and Metric Learning Framework for scDNAm Data

**DOI:** 10.1002/advs.77524

**Published:** 2026-09-01

**Authors:** Yuhang Jia, Siyu Li, Songming Tang, Keju Gu, Shengquan Chen

**Affiliations:** ^1^ School of Mathematical Sciences and LPMC Nankai University Tianjin China; ^2^ College of Life Sciences Nankai University Tianjin China; ^3^ Academy for Advanced Interdisciplinary Studies Nankai University Tianjin China

**Keywords:** cell type annotation, deep metric learning, epigenetic signatures, multi‐scale information, single‐cell DNA methylation

## Abstract

Single‐cell DNA methylation (scDNAm) sequencing provides unique insights into epigenetic heterogeneity and cell‐specific regulatory landscapes. However, accurate cell type annotation for scDNAm data remains challenging, as the distinct data distribution of scDNAm hinders the adaptation of annotation methods from other omics, and specialized annotation tools for scDNAm are currently lacking. Here, MethyAnno is proposed as an interpretable deep metric learning framework that leverages multi‐scale information for accurate cell type annotation of scDNAm data. Additionally, MethyAnno enables generalized category discovery in open‐set scenarios by utilizing density‐based clustering to automatically estimate the number of novel cell types, while simultaneously deciphering cell‐type‐specific epigenetic signatures for biological interpretability. Extensive experiments demonstrate that MethyAnno excels in cross‐dataset annotation and novel type discovery, showing exceptional robustness in few‐shot scenarios for rare cell types. Moreover, interpretability analysis in the human brain dataset correctly recovers the genetic link between Sst interneurons and epilepsy heritability, the association of OPC cells with Alzheimer's disease, as well as the regulatory role of Pvalb cells in synaptic plasticity. Taken together, these findings establish MethyAnno as a robust and biologically interpretable tool for accurate cell type annotation and downstream epigenetic analysis.

## Graphical abstract

MethyAnno enables robust and interpretable annotation of single‐cell DNA methylation data by integrating multi‐scale epigenetic information, bidirectional cross‐attention, and prototype‐based metric learning. The framework resolves rare and novel cell types across datasets while revealing cell‐type‐specific epigenetic signatures associated with disease relevance and biological functions.

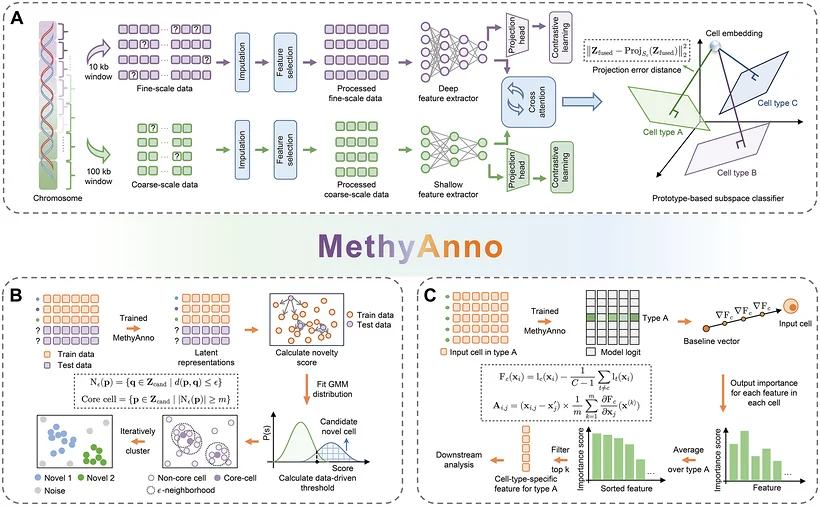

## 1 Introduction

Recent advances in single‐cell sequencing technologies have enabled the characterization of cellular heterogeneity at an unprecedented resolution [1, 2]. This technological foundation has spurred the development of single‐cell epigenomic assays (e.g., scATAC‐seq), which aim to uncover the stable regulatory mechanisms governing cell identity [3, 4, 5]. Among the various epigenetic modifications probed by these assays, DNA methylation is a primary mechanism crucial for cellular regulation. DNA methylation involves the addition of a methyl group to the 5’ carbon of cytosine, typically at CpG dinucleotides, in a reaction catalyzed by DNA methyltransferases [6]. The process of DNA methylation is essential for regulating gene expression, maintaining genome stability, and guiding cell differentiation [6, 7]. Therefore, single‐cell DNA methylation (scDNAm) profiling provides an unparalleled opportunity to dissect methylation heterogeneity and deepen the understanding of cell‐specific regulatory landscapes [4, 8, 9, 10].

Accurate cell type annotation is a fundamental prerequisite for many downstream analyses of scDNAm data, including identifying differentially methylated regions and reconstructing developmental trajectories [4, 9]. While the conventional annotation workflow relies on unsupervised clustering followed by manual annotation with prior biological information, a process that is time‐consuming and lacks reproducibility, the fields of scRNA‐seq and scATAC‐seq have gradually shifted towards automated annotation methods [11, 12, 13]. In these modalities, cell type annotation methods based on machine learning and deep learning are now widely adopted, significantly improving annotation speed and consistency [12, 13, 14, 15, 16, 17, 18]. The success of these approaches suggests a promising avenue for automating cell type annotation in scDNAm data, offering the potential to substantially improve analysis efficiency and generate reproducible results.

However, realizing this potential is far from straightforward. Currently, there is no automated cell type annotation method specifically designed for scDNAm data. While established annotation methods developed for scRNA‐seq and scATAC‐seq are technically applicable to scDNAm data, directly adapting them poses considerable challenges due to several inherent limitations: (1) Annotation methods designed for other modalities are fundamentally challenged by the unique scDNAm data structure, which is not only extremely high‐dimensional but, when aggregated into regional average methylation levels, is also continuously distributed between 0 and 1 [19, 20]. (2) Current methods show poor performance in important, practical cross‐dataset cell type annotation experiments. (3) The intrinsic class imbalance of biological tissues biases models toward majority populations, which hinders the accurate annotation of rare cell types that are often essential for defining overall tissue function and state. (4) Operating under a flawed “closed‐set” assumption, current tools lack the capability to identify novel cell types present in a query dataset but absent from the reference, thereby preventing the characterization of previously unrecognized cellular heterogeneity. (5) Many existing methods lack interpretability, a “black box” nature that prevents researchers from understanding the key cell‐type‐specific epigenetic features driving a classification, thus hindering the understanding of the underlying biological basis of cell identity.

To address these diverse challenges, we present MethyAnno, an interpretable framework for scDNAm data leveraging multi‐scale feature fusion and prototype‐based metric learning for accurate cell type annotation especially for cross‐dataset scenarios. MethyAnno first constructs multi‐scale information from scDNAm profiles and employs an asymmetric dual‐branch network for feature extraction, with a cross‐attention mechanism facilitating fusion of the dual‐branch features to generate unified cell embeddings. Subsequently, MethyAnno leverages a prototype subspace classifier within a deep metric learning framework, where each cell type is represented by an independent subspace composed of multiple basis vectors. We rigorously evaluated MethyAnno across a wide array of challenging cross‐dataset scenarios. In these comprehensive experiments, MethyAnno consistently demonstrated superior performance and robustness, significantly outperforming baseline methods, demonstrating exceptional accuracy even in few‐shot learning tasks involving rare cell types.

Beyond classification, MethyAnno identifies potential novel cell types by measuring the distance of test cells from the embedding spaces of known cell types. Crucially, instead of simple binary detection, MethyAnno leverages density distribution analysis to estimate the specific number of distinct novel cell types. Furthermore, MethyAnno supports interpretability by identifying scDNAm features specific to each cell type. By applying the interpretability framework to the human brain middle temporal gyrus scDNAm dataset, we validated the biological significance of the identified cell‐type‐specific features, uncovering critical insights such as the genetic link between Sst interneurons and epilepsy heritability, the association of OPC cells with Alzheimer's disease, and the regulatory role of Pvalb cells in synaptic plasticity. By bridging computational predictions with these biological mechanisms, MethyAnno establishes itself as a robust and biologically interpretable annotation tool.

## 2 Results

### 2.1 The Overview of MethyAnno

**FIGURE 1:**
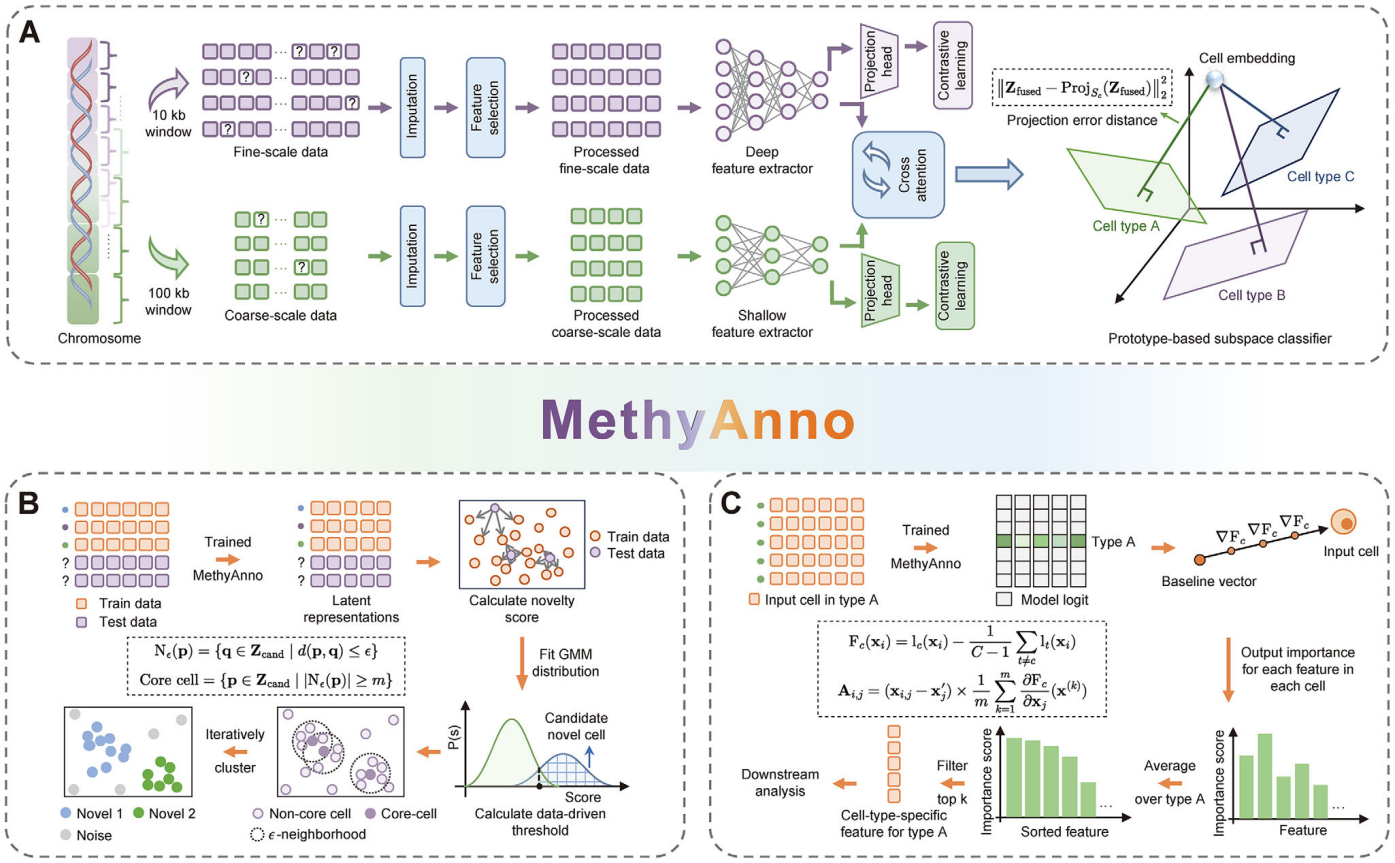
The workflow of MethyAnno. (A) MethyAnno first constructs multi‐scale single‐cell DNA methylation (scDNAm) data and performs preprocessing, then uses an asymmetric dual‐branch network for feature extraction. After feature fusion via cross‐attention, a prototype‐based subspace classifier based on metric learning is applied for classification. (B) MethyAnno quantifies the novelty score of each test cell by calculating the distance to its *k* nearest neighbors in the training set within the latent space, then performs data‐driven threshold calculation, and finally isolates novel cell types by expanding from high‐density core cells. (C) MethyAnno uses a gradient‐based method to quantify the importance of each input feature for explaining the model's output, and the resulting cell‐type‐specific features can be applied to a series of downstream analyses.

The MethyAnno framework consists of four main stages: multi‐scale information construction and preprocessing, feature extraction and cell type annotation, novel cell type identification, and interpretability analysis (Figure 1). The first stage of the MethyAnno framework is multi‐scale information construction and preprocessing. Given the cell‐by‐genomic‐region scDNAm matrix (Methods), we define this raw matrix as the fine‐scale information capable of resolving fine‐grained local epigenetic variations. From this fine‐scale information, a complementary coarse‐scale information is generated via fixed‐window averaging across genomic bins per chromosome to smooth technical noise and capture global patterns, followed by data preprocessing on both information components (Methods).

To extract discriminative features from the multi‐scale inputs, MethyAnno employs an asymmetric dual‐branch network to simultaneously capture complementary epigenetic signals from both fine‐ and coarse‐scale information, ensuring a comprehensive characterization of cellular heterogeneity (Methods). The fine‐scale information is processed by a high‐capacity deep feature extractor to resolve subtle local variations distinguishing closely related cell types, while a low‐capacity shallow feature extractor handles coarse‐scale information to learn noise‐resistant smoothed representations. While a projection head is appended to each branch to generate compressed representations for supervised contrastive learning, the subsequent feature fusion stage operates directly on the representations output by these feature extractors. These multi‐scale representations are then integrated via a bidirectional cross‐attention mechanism [21], where representations from each scale serve as queries to extract context from the opposing scale's keys and values (Methods). Then the two resulting refined representations are concatenated to yield a unified cellular embedding.

Subsequently, the unified cellular embedding is projected into a prototype‐based metric learning framework for cell type identification (Methods). Unlike traditional metric learning approaches that map classes to single points in the latent space, MethyAnno represents each cell type as a learnable low‐dimensional subspace spanned by a set of basis vectors. By learning the underlying structural variance of a cell type and modeling the intra‐class manifold structure, this prototype subspace classifier becomes highly resilient to data shifts from both technical and biological sources while ensuring robust identification of rare cell types in few‐shot settings. Specifically, MethyAnno determines cell type by minimizing the projection error distance between the cell embedding and each subspace, with the entire framework jointly optimized via a multi‐task objective function (Methods). Through comprehensive ablation studies, we systematically evaluated the contribution of the designed multi‐scale information, the cross‐attention mechanism for feature fusion, the prototype‐based subspace classifier, and the various loss components (Text S1 and Table S1). Furthermore, to enhance reproducibility and transparency, all hyperparameters utilized throughout the MethyAnno pipeline have also been detailed in Table S2.

Beyond accurate classification, MethyAnno facilitates generalized category discovery (GCD) to overcome the “closed‐set” limitation (Methods). The framework first identifies potential novel candidates using a novelty score and a data‐driven threshold derived from a two‐component Gaussian mixture model (GMM), where cells exceeding this threshold are regarded as potential novel candidates. Subsequently, MethyAnno employs density‐based clustering to organize these candidates. Unlike graph‐partitioning algorithms such as Louvain [22] and Leiden [23] that force all cells into clusters, the density‐driven approach selectively isolates coherent high‐density regions while effectively filtering out noise. We first identify core cells located in high‐density regions via sufficient local neighbors, then iteratively expand these core cells with their neighbors to form clusters. Crucially, this approach facilitates GCD [24]: rather than grouping unknown cells into a single entity, MethyAnno leverages density distributions to automatically estimate the number of novel cell types and partition cells into distinct sub‐types.

In addition to novel type discovery, MethyAnno performs a feature attribution analysis on the trained model to quantify the average contribution of each genomic region to a specific cell type (Methods). For each cell type, we define a contrastive score that quantifies differential activation between target and non‐target cell type predictive logits. The score is attributed to each feature by accumulating gradients along a path from baseline to the input cell, averaged across all cells belonging to the target cell type to generate final feature importance scores. The interpretability analysis in MethyAnno yields a ranked list of the most discriminative genomic regions, allowing for the identification of key cell‐type‐specific features for downstream analysis.

### 2.2 MethyAnno Demonstrates Accurate Annotation in Diverse Human and Mouse Brain Regions Across Donors and Technologies

To evaluate MethyAnno's cross‐dataset annotation performance, we first conducted a series of experiments on datasets sourced from the same anatomical region but distinct donors. To ensure robust validation, we collected these multi‐sample scDNAm datasets from different species, tissues, and anatomical regions (Methods, Table S3). Specifically, we collected datasets from five mouse brain regions and five human brain regions [4, 8, 9], obtaining a total of ten datasets for each species by ensuring every anatomical region was represented by two distinct donors. To extend tissue diversity, we further included a human adipose tissue dataset comprising five donors [25].

As there are currently no dedicated cell type annotation tools for scDNAm data, we selected baseline methods that perform well in other single‐cell omics. Recommended by recent benchmark studies for annotation methods in scRNA‐seq [11, 26, 27], we employed representative conventional machine learning algorithms, including support vector machine (SVM), random forest (RF), K‐nearest neighbors (KNN), and XGBoost [28]. To provide a more comprehensive and fair comparison, we evaluated these baseline methods under two settings (Text S2 and Table S4). First, we evaluated the default‐parameter baselines, including K‐nearest neighbors with 9 neighbors or 50 neighbors settings. Second, we performed hyperparameter tuning for all conventional machine learning baselines using five‐fold cross‐validation. The tuned baseline parameters were then fixed and applied to all benchmark experiments. In the main text, we report the results obtained from the tuned baseline models, as this setting provides a fairer comparison with MethyAnno. The results obtained using the default‐parameter baselines are provided in the Supporting Information for transparency.

Moreover, we also benchmarked MethyAnno against MINGLE, a state‐of‐the‐art cell type annotation method for scATAC‐seq [12]. To adapt MINGLE for scDNAm data, we applied two preprocessing strategies (Methods), resulting in two baselines: MINGLE_TF and MINGLE_No_TF. Following recommendations from recent studies [11, 12, 26], model performance was evaluated using three metrics: accuracy (Acc), Cohen's kappa value (Kappa), and macro F1 score (Macro‐F1) (Text S3). Our experimental design employed a region‐specific, leave‐one‐sample‐out validation strategy.

**FIGURE 2:**
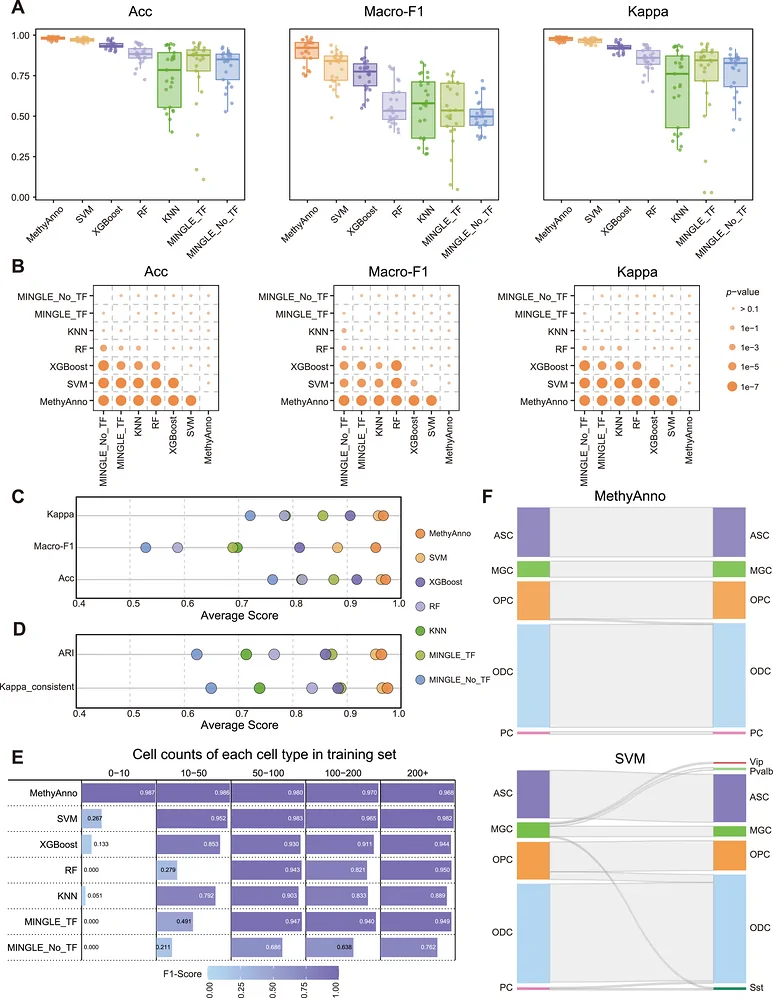
Performance of MethyAnno and baseline methods in cross‐donor and cross‐sequencing technology scenarios. (A) The performance of MethyAnno and baseline methods across 25 intra‐region cross‐donor experiments, presented in box plots. The scatter points represent results of each experimental run. (B) Heatmap of *p*‐values from one‐sided paired Wilcoxon signed‐rank tests determining whether the method in the column achieved significantly higher metrics than the method in the row. (C) The average multi‐class classification metrics of MethyAnno and baseline methods in cross‐sequencing technology experiments. (D) The average consistency metrics of MethyAnno and baseline methods in cross‐sequencing technology experiments. (E) The average F1‐scores of MethyAnno and baseline methods for each cell type stratified by the cell counts in the training set. (F) The annotation results of MethyAnno and SVM for non‐neuronal cell types in the HB‐MTG‐2 dataset, presented in Sankey diagrams.

In the overall results from all cross‐donor experiments, MethyAnno demonstrated superior and consistent performance over all baseline methods (Figure 2A). MethyAnno achieved the highest mean Acc (0.977), Macro‐F1 (0.892), and Kappa (0.972), with SVM consistently performing as the next‐best method. Specifically, MethyAnno surpassed SVM by margins of 0.009 (0.895%) in Acc and 0.010 (1.116%) in Kappa, while achieving a substantial gain of 0.098 (12.403%) in Macro‐F1, a metric that assigns equal weight to every cell type regardless of population size, highlighting the model's balanced performance across all cell types. To statistically quantify MethyAnno's superiority over all baselines, we performed one‐sided paired Wilcoxon signed‐rank tests across a total of 25 experimental trials (Figure 2B). The results confirmed a highly significant advantage: MethyAnno outperformed every baseline method with a *p*‐value less than 1e‐7 across all three metrics. However, the method designed for scATAC‐seq, MINGLE, performed poorly despite our specialized preprocessing (MINGLE_TF and MINGLE_No_TF), yielding low mean accuracy scores (0.777 and 0.803, respectively), which were still superior to the performance without such preprocessing. This finding suggests that the fundamental differences in data modality between scDNAm and scATAC‐seq cannot be resolved by simple data transformation, highlighting the need for a method specifically tailored for scDNAm data. We have also systematically verified the computational efficiency of MethyAnno across 25 cross‐donor annotation experiments within the same anatomical regions (Table S5).

Building on the results, we designed a more challenging scenario to assess MethyAnno's technical robustness: cross‐sequencing technology annotation where the training and test sets originated from the same anatomical region but different sequencing technologies. For this analysis, we collected human brain datasets from the MTG and M1C regions, each represented by two samples generated with snmC‐seq3 and one sample generated with snm3C‐seq (Supplementary Table S3), exhibiting significant batch effects due to the different sequencing technologies (Figure S1). This design yielded eight cross‐sequencing technology annotation tasks, formed by pairing the snm3C‐seq dataset with one of the snmC‐seq3 datasets from the same region and alternating the roles of training and testing sets. As shown in Figure 2C, MethyAnno successfully overcomes the significant technical batch effects introduced by different sequencing platforms. Across both the MTG and M1C regions, MethyAnno achieved exceptionally high mean accuracy scores of 0.983 and 0.959, respectively, reflecting its ability to accurately identify cell types irrespective of the sequencing technology. Consistent with our previous findings, MethyAnno's advantage was still most pronounced in the Macro‐F1 score, confirming that its superior performance in accurately detecting rare cell types is maintained even in the presence of strong technical batch effects.

To further investigate whether MethyAnno learns a technology‐invariant representation, we evaluated the concordance of predictions derived from the cross‐sequencing technology experiments. For each brain region, we compared two specific sets of predictions made on a common snmC‐seq3 test set: (1) the predictions generated by the model trained on the other snmC‐seq3 dataset, and (2) the predictions generated by the model trained on the snm3C‐seq dataset. We then calculated the consistency between these two sets of predictions using the adjusted Rand index (ARI) and a concordance‐specific Kappa score (Kappa_consistent). Figure 2D shows that predictions from the two independently trained MethyAnno models were exceptionally consistent, achieving an average ARI of 0.963 and an average Kappa_consistent score of 0.974 across both brain regions. Such high concordance indicates that MethyAnno captures intrinsic epigenetic identities rather than platform‐specific artifacts, ensuring that biological conclusions remain robust regardless of the sequencing technology. In contrast, the runner‐up method, SVM, exhibited slightly lower stability, averaging an ARI of 0.953 and Kappa_consistent of 0.965. While these scores indicate reasonable stability, MethyAnno's superior performance demonstrates greater robustness against technical variations compared to even the strongest baseline. These results confirm MethyAnno's capacity to learn conserved biological representations, a prerequisite for accurately integrating diverse single‐cell atlases.

### 2.3 MethyAnno Excels in Rare Cell Type Identification and Resolves the Oligodendrocyte Lineage in the Human Brain

As observed in the previous experiments, MethyAnno's most significant advantage was in the Macro‐F1 score. Since Macro‐F1 score assigns equal importance to every class, it provides a rigorous evaluation for scDNAm datasets characterized by natural class imbalance and sparse rare types. MethyAnno's superior Macro‐F1 score directly reflects its distinct ability to accurately identify these rare cell types.

To investigate the underlying cause of MethyAnno's superior Macro‐F1 score, we performed a detailed, per‐class performance analysis on the result of HB‐MTG cross‐donor annotation task (trained on HB‐MTG‐1, evaluated on HB‐MTG‐2). We treated the classification of each cell type as a one‐vs‐rest binary task and calculated the F1‐score, which balances Precision and Recall (Figure 2E). The results revealed that while both MethyAnno and all baseline methods performed well for abundant cell types (over 100 cells), the performance gap became increasingly evident for rare populations. This difference was most evident for extremely rare cell types (less than 10 training cells): while nearly all baseline methods failed to make effective predictions (mean F1‐scores close to 0), MethyAnno maintained a remarkably high F1‐score of 0.987. This finding indicates that MethyAnno can construct discriminative prototype subspaces even in few‐shot scenarios, which is the primary driver of its superior overall performance.

In addition, to conduct a more in‐depth analysis of the performance differences between MethyAnno and all baseline methods, we visualized the detailed classification flows for the non‐neuronal cell lineage for all methods (Figure 2F and Figure S2). Figure 2F reveals a significant annotation failure by the next‐best method, SVM. Compared to MethyAnno, SVM misclassified a larger proportion of OPC cells as the ODC brain cell type and completely misclassified the rare PC cell population. In contrast, MethyAnno was able to clearly and accurately distinguish between the OPC and ODC cell types while successfully identifying the rare PC cells. This precise annotation is biologically critical because mature ODCs differentiate from OPCs, a process that makes the two cell types biologically highly similar and inherently difficult to distinguish, yet functionally distinct [29, 30]. Accurately separating the progenitor (OPC) and mature (ODC) states is essential for investigating the molecular mechanisms underlying central nervous system disorders and development [31, 32]. MethyAnno's success in resolving this challenging lineage highlights its sensitivity to the subtle epigenetic signatures that distinguish closely related cell states.

### 2.4 MethyAnno Robustly Identifies Cell Types Across Anatomical Regions and Captures Region‐Specific Epigenetic Signatures of L2/3 IT Neurons

In the previous experiments, we demonstrated the effectiveness of MethyAnno in cross‐donor annotation experiments within specific anatomical regions. However, large‐scale cell atlas construction often requires annotating samples from unseen anatomical regions based on reference datasets derived from different anatomical regions. To systematically assess MethyAnno's robustness against the biological variability inherent to this realistic generalization scenario, we conducted a series of cross‐dataset experiments between distinct anatomical regions. To this end, we collected comprehensive datasets spanning five human and six mouse brain regions (Methods, Table S3). We subsequently performed all pairwise annotation tasks between different regions within each species.

**FIGURE 3:**
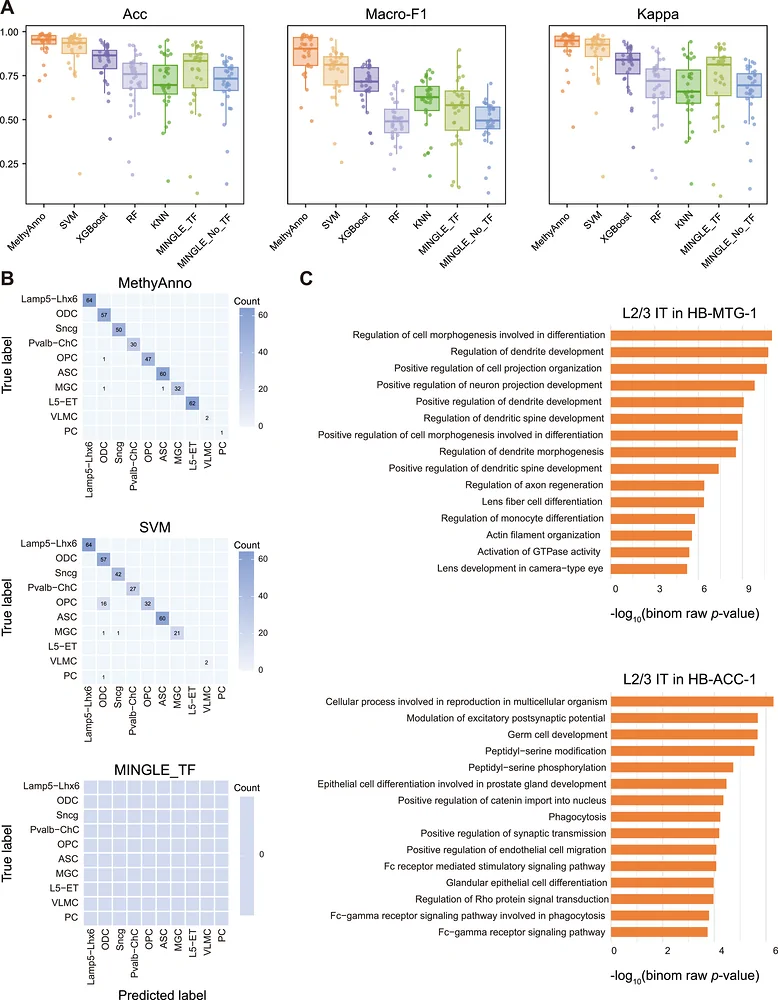
Performance of MethyAnno and baseline methods in cross brain region scenarios. (A) The performance of MethyAnno and baseline methods across all cross‐region experiments, presented in box plots. The scatter points represent results of each experimental run. (B) Prediction results of MethyAnno, SVM, and MINGLE_TF trained on the HB‐MTG‐1 dataset and tested on the HB‐ACC‐1 dataset. Only cell types with fewer than 50 training cells are visualized, where values represent column cell types predicted as row cell types. (C) Enrichment results of GREAT analysis for cell‐type‐specific features detected by MethyAnno, displaying the top 15 significantly enriched biological processes.

The results across all cross‐region experiments confirm MethyAnno's capability to overcome inter‐regional epigenetic heterogeneity and accurately transfer cell type labels across anatomical boundaries (Figure 3A). MethyAnno consistently performed best across all metrics, surpassing the next‐best method, SVM, with relative gains of 4.676% in Acc, 5.129% in Kappa, and a substantial 11.923% in Macro‐F1. The boxplots demonstrate that MethyAnno achieved stable performance with the narrowest interquartile range, whereas baseline methods like KNN, MINGLE_TF, and MINGLE_No_TF were highly unstable with significantly wider variance. Notably, in specific cross‐region combinations, Acc score for these three baseline methods fell below 0.25, indicating a failure to capture conserved biological signatures across anatomical regions. Consistent with the within‐region tests, MethyAnno's most significant performance gap remained in the Macro‐F1 score, demonstrating its superior capacity to resolve rare cell types that are frequently overshadowed by major cell types in cross‐region scenarios.

To explore annotation performance on rare cell types in detail, we performed a case study on the human cerebral cortex, where models were trained on the middle temporal gyrus (HB‐MTG‐1) and tested on the anterior cingulate cortex (HB‐ACC‐1). Here, we focused the analysis on cell types with fewer than 50 training cells (Figure 3B and Figure S3). The confusion matrix heatmap of MethyAnno's prediction results displays a distinct diagonal, indicating accurate classification of most rare types. In contrast, SVM, even as the next‐best method, produced scattered distributions and failed to annotate any rare types correctly. This failure aligns with SVM's inherent limitations: its decision boundary relies on support vectors, and sparse samples from rare cell types lead to unstable boundaries easily biased by dominant neighboring types. MINGLE_TF showed an even more severe annotation failure: failing to correctly predict any cells for the visualized rare types, which directly explains its low Macro‐F1 score in the previous experiments.

Finally, we investigated whether MethyAnno learned both conserved biological features representing the core identity of shared cell types and region‐specific epigenetic signatures across different anatomical regions. We focused on the L2/3 IT type, a major neuronal type present in both the HB‐MTG‐1 and HB‐ACC‐1 datasets. MethyAnno quantifies the importance of genomic regions by calculating average attribution scores within the trained model. Using MethyAnno's interpretability module, we identified the top 1000 cell‐type‐specific features for L2/3 IT in each dataset and used the gene regulatory enrichment analysis tool (GREAT) (Text S4) to identify the significant biological processes associated with these region‐specific features of L2/3 IT cell type. First, as shown in Figure 3C, the analysis confirmed the fundamental identity of L2/3 IT neurons. Results in both the MTG and ACC regions converged on neuronal structure‐ and synaptic‐function‐related processes, which aligns with their established role as pyramidal neurons that send major axonal projections, a process reliant on their complex dendritic and synaptic structures [33, 34].

More notably, the results of GREAT analysis also revealed distinct, region‐specific biological terms. For the MTG‐derived L2/3 IT feature set, we detected significant enrichment for “regulation of dendritic spine development” and “actin filament organization”. This finding aligns with the role of MTG in memory processing [35, 36], given that dendritic spines are actin‐rich postsynaptic structures whose remodeling is a key component of the synaptic plasticity underlying long‐term memory formation [37, 38]. Conversely, the ACC‐derived L2/3 IT feature set showed a coherent immune‐related enrichment pattern, including “Fc receptor mediated stimulatory signaling pathway” and “Fc‐gamma receptor signaling pathway involved in phagocytosis”. This signature aligns with the ACC's established function in regulating pain‐associated emotional states [39, 40]. Mechanistically, chronic pain can induce neuroinflammatory remodeling in the ACC, where microglial activation and inflammatory signaling can alter neuronal excitability and synaptic transmission and contribute to negative affect [41]. In this context, the Fc‐receptor and phagocytosis‐related enrichment suggests that MethyAnno captures a neuroimmune‐associated regulatory signature relevant to the ACC's involvement in pain processing. Taken together, these findings demonstrate that MethyAnno not only ensures superior accuracy and robustness by successfully capturing the fundamental characteristics of a cell type, but also serves as a powerful biological discovery tool capable of revealing context‐specific biological insights.

### 2.5 MethyAnno Uncovers Epigenetic Links to Epilepsy Heritability in Sst Interneurons and Delineates Functional Regulatory Landscapes in the Human Middle Temporal Gyrus

To further dissect the molecular landscape of the human middle temporal gyrus, we applied MethyAnno's interpretability framework to the HB‐MTG‐1 dataset, which comprises a complex heterogeneity of telencephalic excitatory neurons, inhibitory neurons, and non‐neuronal cell types. Specifically, we selected the top 1,000 features in raw data with the highest attribution scores for each cell type in the HB‐MTG‐1 dataset to serve as cell‐type‐specific features. We then performed a series of downstream analyses (Text S4) to validate the biological relevance of these features compared to the background features.

**FIGURE 4:**
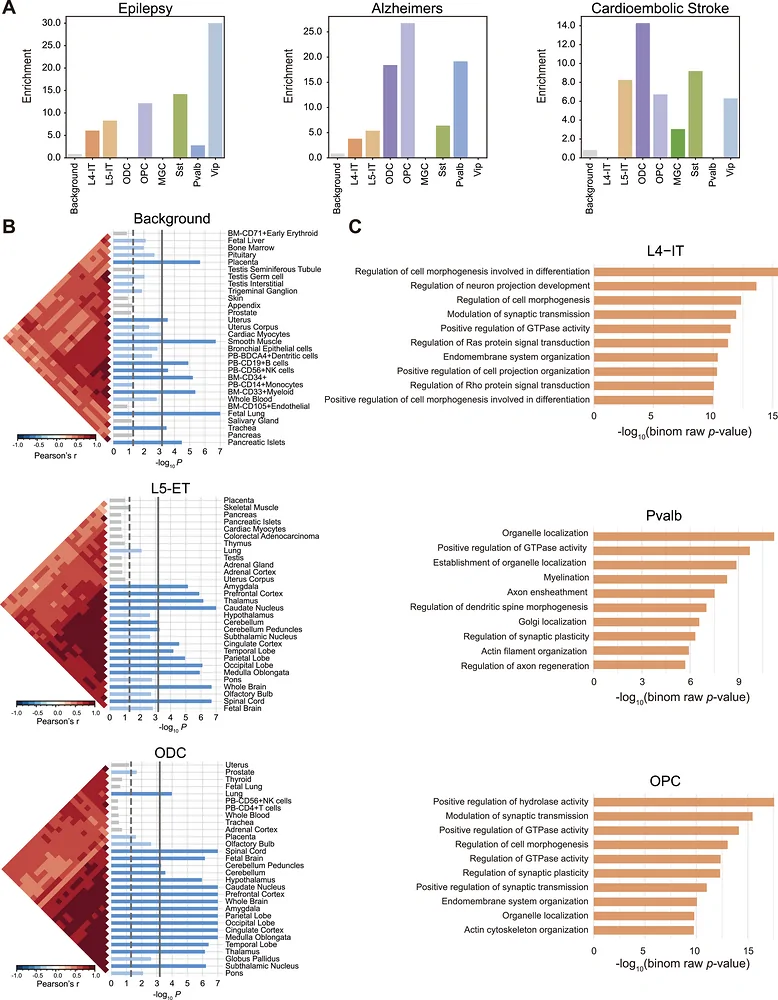
Downstream analyses of cell‐type‐specific features identified by MethyAnno. (A) Heritability enrichment of three neurological and psychiatric disorders, estimated via LDSC using cell‐type‐specific features identified by MethyAnno relative to background features. (B) Top 30 tissues showing significant enrichment in SNPsea analysis performed on MethyAnno‐identified cell‐type‐specific features and background features. (C) GREAT analysis results for cell‐type‐specific features identified by MethyAnno, illustrating the top 10 significantly enriched biological processes.

First, the cell‐type‐specific features identified by MethyAnno exhibit meaningful associations with neurological and brain‐related diseases. To assess this genetic relevance, we quantified the heritability enrichment of three major neurological and brain‐related diseases within each set of cell‐type‐specific genomic regions and the background regions set using partitioned linkage disequilibrium score regression (LDSC) [42] with default settings. Explicitly, this background feature set is generated by excluding the union of all cell‐type‐specific features across all cell types from the whole‐genome feature set. As shown in Figure 4A, multiple cell types in the HB‐MTG‐1 dataset exhibited significant enrichment compared to the background features for the tested diseases. Notably, the Sst cell type exhibited a significant heritability enrichment for epilepsy. Prior research has extensively documented the critical link between this cell type and the disease, specifically demonstrating that seizures induce the selective loss of Sst‐expressing interneurons, which may constitute a key pathological mechanism in epilepsy [43, 44, 45]. Furthermore, the enrichment analysis indicated that OPC cells are significantly associated with Alzheimer's disease, highlighting the potential involvement of these cell types in the disease's pathogenesis.

Second, the cell‐type‐specific features identified by MethyAnno demonstrate tissue‐specific expression enrichment. We performed SNPsea analysis [46] with default settings to evaluate the specificity of the cell‐type‐specific features and background features across 79 distinct human tissues. We illustrated the top 30 significantly enriched tissues in Figure 4B. The results showed that compared with background features, both L5‐ET and ODC cell types exhibited more pronounced enrichment in brain and nervous system‐related tissues. Furthermore, the tissue‐specific enrichment profiles accurately reflected the distinct anatomical distributions of L5‐ET and ODC cell types. While L5‐ET neurons are excitatory projection neurons distributed in the cortex [33], ODC cells are widely distributed glial cells distributed throughout the central nervous system [47]. Consistent with these anatomical features, compared to the results of L5‐ET neurons, ODC‐specific features demonstrated significantly stronger enrichment in subcortical structures, such as the hypothalamus and subthalamic nucleus.

Finally, the cell‐type‐specific features identified by MethyAnno can capture the functional characteristics of corresponding cell types. We performed GREAT analysis [48] on the identified features to uncover significant biological processes associated with each cell type and displayed the top 10 biological processes for each cell type in Figure 4C. The results demonstrated that the cell‐type‐specific features for L4‐IT cells are associated with processes governing neural connectivity, including “regulation of neuron projection development” and “modulation of synaptic transmission”. This is consistent with the known biological function of L4‐IT cells in establishing intracortical projections [33]. Furthermore, the association between Pvalb cells and “regulation of synaptic plasticity” was highly significant, which aligns with the role of Pvalb cells in precisely modulating the synaptic plasticity of excitatory neurons through rapid inhibitory synaptic transmission [49]. The results also revealed that the biological processes identified for OPCs are closely related to cell morphogenesis and differentiation regulation, such as “regulation of cell morphogenesis”. Collectively, these downstream analyses validate that the cell‐type‐specific features identified by MethyAnno are not only statistically meaningful but also biologically relevant, providing interpretable insights into the association with relevant diseases, tissue specificity, and functional characteristics of each cell type.

### 2.6 MethyAnno Effectively Identified Novel Cell Types

In practical applications of cell type annotation, models frequently encounter “open‐set” scenarios where the test data contains cell types absent from the training set [50]. These unseen cell types, referred to as novel cell types, often represent rare subtypes, transitional states, or distinct biological discoveries. Therefore, the capacity to identify novel cell types is a critical requirement for automated annotation tools.

To evaluate MethyAnno's capability in novel type discovery, we maintained the experimental setup from the previous section, using the dataset HB‐MTG‐1 for training and the dataset HB‐MTG‐2 for testing. To simulate the presence of novel types, we removed specific cell types from the training set while retaining them in the test set. Here, we benchmarked MethyAnno against SVM with a rejection option (SVMrejection), a method previously recommended for novel type detection in scRNA‐seq data in a benchmark study [26]. Additionally, we compared MethyAnno with MINGLE, which incorporates a specific module for novel type detection based on convex hull geometry [12].

**FIGURE 5:**
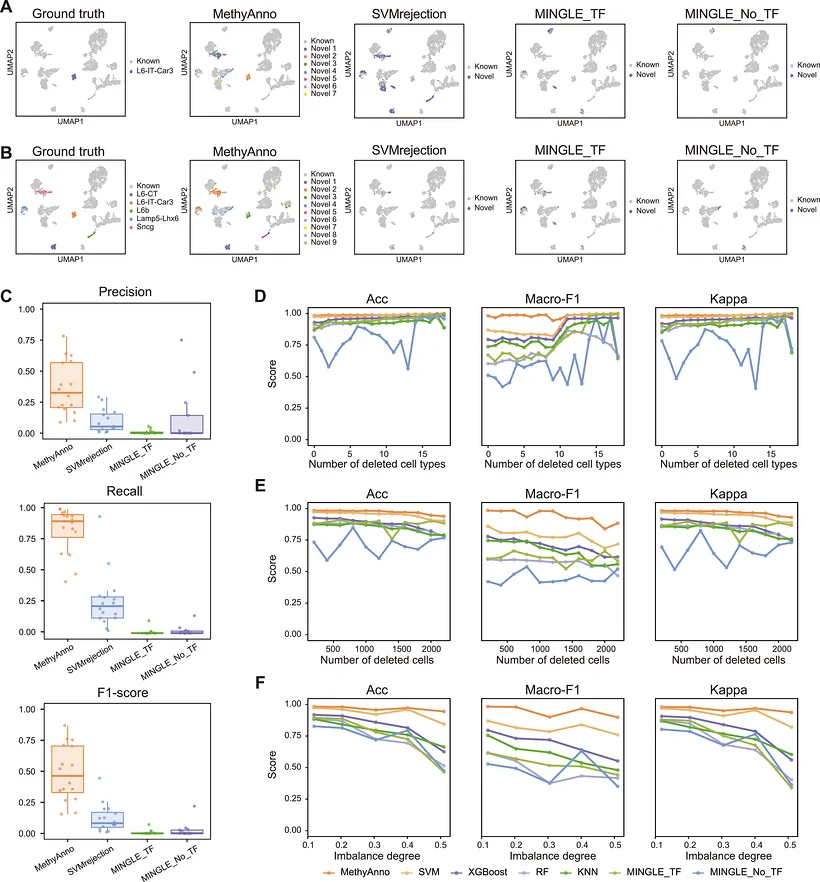
The results of novel type discovery and robustness of MethyAnno. (A,B). UMAP visualizations of the HB‐MTG‐2 test set (reduced to 50 dimensions via PCA), illustrating the distribution of simulated novel types and the prediction results of MethyAnno and baseline methods. The scenarios include a single novel cell type (A) and five novel cell types (B). (C). Performance of MethyAnno, SVMrejection, and MINGLE in novel cell type identification. Each data point in the box plots represents an experiment where a single specific cell type was excluded from the training set to simulate a novel class. (D–F). Robustness analysis of MethyAnno and baseline methods under varying data configurations. The plots display performance trends with respect to the number of deleted cell types (D), the number of deleted cells (E), and the degree of class imbalance (F).

We first assessed the performance of MethyAnno in identifying a single novel cell type. We selected L6‐IT‐Car3 as the novel type and excluded it from the training set. L6‐IT‐Car3 is a rare excitatory neuronal population consisting of intratelencephalic projection neurons in cortex layer 6 and neurons from the claustrum. Due to its scarcity and distinct spatial origin, it serves as an ideal candidate for a novel type of simulation. We performed principal components analysis (PCA) dimensionality reduction on the test set and visualized the identification results via UMAP projection (Figure 5A). Note that the UMAP visualization is used to display the successful identification of novel cell types and the filtering of noise, rather than representing biological differentiation paths. The UMAP visualization explicitly displayed the successful identification of L6‐IT‐Car3 cells, confirming the model's capability to detect novel cell types. In the annotation results, although MethyAnno exhibited minor misclassifications of known types, it successfully identified a substantial proportion of the authentic L6‐IT‐Car3 cells. In contrast, the baseline methods failed to achieve effective novel cell type recognition. SVMrejection exhibited a high false‐positive rate, erroneously flagging numerous known cells as novel. Meanwhile, both MINGLE_TF and MINGLE_No_TF failed to detect the authentic novelty of L6‐IT‐Car3 cells, completely misclassifying them as known types.

To further verify MethyAnno's novelty detection performance in more complex scenarios, we designed an experiment to identify multiple novel cell types. We removed five rare cell types from the training set (L6‐CT, L6‐IT‐Car3, L6b, Lamp5‐Lhx6, and Sncg), and treated them as novel types. As shown in Figure 5B, MethyAnno demonstrated its robust capability for GCD by not only successfully recognizing most cells of novel types but also automatically estimating the number of distinct novel populations. It partitioned these correctly recognized novel cells into seven independent clusters, closely aligned with the ground truth biological heterogeneity. While MINGLE_TF identified a small fraction of correct novel cells, the other baselines produced negligible detection results, indicating their inability to effectively distinguish multiple unseen cell types from the background of known types.

Finally, to ensure statistical robustness, we conducted a more comprehensive evaluation. We iterated through every cell type in the HB‐MTG‐1 dataset, removing one type at a time from the training set to serve as the novel type. We treated novel type identification as a binary classification task (novel vs. known) and calculated Precision, Recall, and F1‐score for each iteration. As shown in Figure 5C, MethyAnno significantly outperformed all comparative methods, achieving a mean Recall of 0.828, a Precision of 0.365, and an F1‐score of 0.484. Even the second‐best performer, SVMrejection, only achieved an average Recall of 0.256, with a mean Precision of 0.095 and an F1‐score of 0.121. Both MINGLE variants (MINGLE_TF and MINGLE_No_TF) exhibited even poorer performance, yielding Recall scores below 0.1 and more negligible Precision and F1‐scores, suggesting that these methods are ineffective for identifying novel types.

### 2.7 MethyAnno Maintains Stable Performance Under Varying Data Conditions

Single‐cell DNA methylation datasets exhibit significant heterogeneity in terms of the number of cell types, data size, and imbalance degree. Consequently, the robustness and stability of an annotation framework across diverse data configurations are fundamental indicators of its practical utility. In this section, we performed a systematic robustness evaluation between MethyAnno and baseline methods, consistently utilizing HB‐MTG‐1 as the training set and HB‐MTG‐2 as the test set, creating a series of experimental scenarios to assess model performance under varying constraints.

We first investigated model stability relative to the number of cell types present in the dataset. We generated artificial datasets by iteratively removing cell types from both training and testing sets until only three classes remained, thereby ensuring the evaluation was conducted within a multi‐class classification framework. Figure 5D presents the performance metrics across different numbers of deleted cell types. MethyAnno consistently demonstrated superior performance compared to all baseline methods, maintaining high accuracy and stability regardless of the number of cell types. Conversely, MINGLE_No_TF exhibited the lowest performance across all metrics. This aligns with our previous observations, reinforcing the conclusion that simplistic data transformation is insufficient to adapt scATAC‐seq‐specific methodologies to scDNAm data. Furthermore, MINGLE_No_TF displayed significant variance in the results, which suggests that MINGLE_No_TF is highly dependent on the composition of cell types, and unable to learn generalized discriminative features with changes in the number of cell types.

Next, we evaluated the sensitivity of each method to training set size, simulating scenarios of limited data availability. We repeatedly performed global downsampling on the training set in steps of 200 cells until the total number of cells fell below 200, while maintaining a constant cell type composition in the test set. As shown in Figure 5E, MethyAnno maintained stable performance, consistently ranking as the top method with only slight degradation even in data‐limited scenarios, while both MINGLE_TF and MINGLE_No_TF exhibited substantial metric fluctuations. Moreover, we noticed that the KNN method displayed a monotonic decrease in performance as the dataset size decreased. This trend can be attributed to the fixed *k* parameter in the K‐Nearest Neighbors algorithm: as the size of the training set decreases, the algorithm inevitably includes neighbors from different cell types to satisfy the fixed count. This introduces noise into the voting process, thereby blurring decision boundaries and reducing the classification accuracy of KNN.

Finally, given that scDNAm datasets frequently exhibit varying degrees of imbalance, manifesting as a long‐tail distribution driven by the presence of numerous rare cell types, we assessed the robustness of MethyAnno against various degrees of class imbalance, which is defined by estimating the normalized entropy of the cell type size distribution in the dataset (Text S3). We generated five artificial datasets with imbalance degrees ranging from 0.119 to 0.510 by randomly downsampling specific cell types. A higher imbalance degree indicates a stronger distribution bias toward majority classes. Figure 5F illustrates that while baseline methods suffered a marked performance decline as imbalance increased, MethyAnno remained stable. Specifically, MethyAnno maintained a Macro‐F1 score exceeding 0.9 across all imbalance levels, which indicates that MethyAnno effectively extracts representative features for both majority and minority classes. Conversely, the significant performance drop in almost all baseline methods suggests an inability to learn adequate representations from underrepresented classes, leading to poor accuracy on rare cell types.

Taken together, these extensive evaluations underscore the exceptional robustness of MethyAnno across diverse and challenging data configurations. Unlike baseline methods that are prone to performance degradation under conditions of data scarcity, reduced number of cell types, or severe distributional imbalance, MethyAnno maintains consistent and superior accuracy. This resilience confirms MethyAnno's reliability and generalizability, making it highly suitable for the complex and heterogeneous nature of real‐world single‐cell DNA methylation studies.

## 3 Discussion

Single‐cell DNA methylation sequencing offers unique insights into the epigenomic landscape, yet accurately annotating these datasets remains a critical bottleneck. There is a distinct lack of annotation tools specifically designed for scDNAm data. Furthermore, due to the unique data distribution characteristics of the scDNAm modality, direct migration of methods developed for other modalities, such as scRNA‐seq and scATAC‐seq often proves ineffective. In this study, we presented MethyAnno, a framework designed to address this gap by prioritizing robustness across heterogeneous datasets and interpretability of biological features. Our extensive experiments confirm that MethyAnno effectively mitigates domain shifts and accurately identifies rare cell types, providing a reliable tool for analyzing complex scDNAm data. Specifically, our interpretability analysis uncovered biologically meaningful insights, exemplified by the genetic link between Sst interneurons and epilepsy, the association of OPC cells with Alzheimer's disease, and the regulatory role of Pvalb cells in synaptic plasticity.

Despite MethyAnno's superior performance, there are several avenues for further improvement. First, MethyAnno currently relies solely on scDNAm data. Given the abundance of well‐annotated scRNA‐seq atlases, future iterations could explore transfer learning or multi‐modal integration to leverage transcriptomic knowledge for enhancing epigenetic annotation. Second, MethyAnno focuses on dissociated single cells, potentially overlooking cell‐cell interactions. Extending the framework to accommodate emerging spatial epigenomics data would enable the characterization of cell types within their native tissue microenvironment. Third, the current novelty detection strategy may be further improved in more complex open‐set scenarios. For example, cells on a differentiation continuum may show gradual transitions between known and novel states, and known cell types with strong intra‐type heterogeneity or donor‐related shifts may have variable distances to the training manifold. Future work could explore more flexible novelty detection strategies, such as local thresholds, data‐driven mixture modeling, or trajectory‐aware methods, to improve robustness in these cases. Finally, the current training paradigm is static. Incorporating continuous learning (or online learning) capabilities would allow the model to incrementally update itself with newly sequenced batches or donors without requiring full retraining, making it more scalable for expanding cell atlases.

## 4 Conclusion

In this paper, we propose MethyAnno, an interpretable deep learning framework tailored for accurate cell type annotation of scDNAm data. By integrating a multi‐scale asymmetric network with a prototype subspace classifier, MethyAnno effectively addresses the challenges of data heterogeneity and class imbalance. Through a series of cross‐dataset experiments, including cross‐donor and inter‐region tasks, we demonstrate that MethyAnno significantly outperforms conventional machine learning methods and state‐of‐the‐art methods designed for scATAC‐seq. Notably, MethyAnno exhibits exceptional performance on rare cell types and successfully identifies novel cell populations in open‐set scenarios by combining local distance metrics with density‐based clustering. Furthermore, our interpretability analyses confirm that the genomic features learned by MethyAnno are biologically meaningful, correctly recovering the genetic basis of neurological diseases, capturing tissue‐specific expression enrichments, and revealing the biological processes associated with the essential functions of cell types.

MethyAnno offers the following key advantages: (1) MethyAnno employs an asymmetric dual‐branch feature extraction network that effectively captures epigenetic signatures while filtering out technical noise. (2) MethyAnno designs a prototype‐based metric learning strategy to model cell types as learnable subspaces, ensuring robust alignment across different datasets and precise identification of rare cell types. (3) MethyAnno incorporates an adaptive density‐based clustering mechanism to effectively identify and separate novel cell populations absent from the training reference. (4) MethyAnno provides biological interpretability by pinpointing cell‐type‐specific genomic regions that reveal the genetic basis of diseases and essential cellular functions.

## 5 Methods

### 5.1 The Overview of MethyAnno

After multi‐scale information construction and preprocessing for the training and test sets, MethyAnno first utilizes an asymmetric dual‐branch feature extractor to derive feature representations from both fine‐scale and coarse‐scale information. Then, we apply a cross‐attention mechanism to fuse the features from these two branches, yielding a unified embedding for each cell. To perform classification, we learn a prototype subspace representation for each cell type in the training set and feed the cell embeddings into a metric‐based prototype classifier, which determines the cell type label by calculating the projection error to each prototype subspace. Moreover, MethyAnno integrates a novelty identification module based on cell distance and density clustering to detect potential new cell types that may appear in the test set. Finally, to explain the trained model's predictions, we design a gradient‐based method to calculate importance scores for each input feature, thereby identifying cell‐type‐specific features that facilitate various downstream analyses. A detailed description of all model hyperparameters and their exact values is provided in Table S2.

### 5.2 Model Structure—Asymmetric Dual‐Branch Feature Extractor

MethyAnno utilizes an asymmetric dual‐branch network that takes two distinct cell‐by‐region matrices for feature extraction: a fine‐scale preprocessed data matrix $X_{\mathrm{fine}}\in R^{N\times D_{1}}$ and a coarse‐scale preprocessed data matrix $X_{\mathrm{coarse}}\in R^{N\times D_{2}}$. Both branches are constructed using a standardized building block comprising a Linear layer, Batch Normalization, GeLU activation, and Dropout. The fine‐scale branch employs a deeper architecture consisting of three sequential blocks, which progressively reduce the feature dimension from *D*_1_ to 1024, 512, and finally 256 to generate the embedding $Z_{\mathrm{fine}}\in R^{N\times 256}$. In parallel, the coarse‐scale branch utilizes a shallower architecture with two blocks, reducing dimensions from *D*_2_ to 512 and then 256, yielding the embedding $Z_{\mathrm{coarse}}\in R^{N\times 256}$. Additionally, a projection head is attached to each branch to map the embeddings through a two‐layer network to a 128‐dimensional space. The resulting outputs, 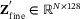 and 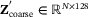, are L2‐normalized and utilized for the contrastive learning objective.

### 5.3 Model Structure—Cross‐Attention Mechanism for Feature Fusion

To integrate information from both branches, we employ a bidirectional cross‐attention module. We detail the update mechanism for the fine‐scale features **Z**_fine_ using coarse‐scale features **Z**_coarse_ as context while the reverse operation is symmetrical. First, the 2D input feature matrices **Z**_fine_ and **Z**_coarse_ are transformed into 3D sequence tensors by projecting each feature value into a high‐dimensional embedding space via a linear layer, expanding the dimensions to $R^{N\times 256\times d_{\mathrm{model}}}$ (where *d*_model_ =  64). Learnable index‐specific encodings $E_{\mathrm{pos}}\in R^{256\times d_{\mathrm{model}}}$ are added to these embeddings to retain feature‐specific positional information, and we denote these 3D position‐aware sequences as **S**_fine_ and **S**_coarse_ respectively. Layer normalization (LN) is applied to these feature sequences. Subsequently, the attention mechanism is computed. MethyAnno utilizes a bidirectional cross‐attention mechanism to integrate multi‐scale information. For the fine‐scale update, the query (**Q**) is derived from **S**_fine_, while the key (**K**) and value (**V**) are derived from **S**_coarse_. In this operation, the attention mechanism systematically captures the dependencies between local and global genomic scales, utilizing the global epigenetic landscape as a comprehensive contextual reference to enhance the representation of localized fine‐grained methylation signals. Then we use attention operation to compute the context‐aware representations:

$$\begin{array}{ccc} H_{\mathrm{attn}} & = & \;\mathrm{Attention}\left(Q=\;\mathrm{LN}\left(S_{\mathrm{fine}}\right),K=\;\mathrm{LN}\left(S_{\mathrm{coarse}}\right),\right.\\ V & = & \left.\;\mathrm{LN}\left(S_{\mathrm{coarse}}\right)\right) \end{array}$$

The attention output **H**_attn_ is combined with the original query sequence via a residual connection, followed by a feed‐forward network (FFN) with a second residual connection. Finally, a linear layer projects the *d*_model_‐dimensional embeddings back to scalars, restoring the original tensor shape $R^{N\times 256}$. An analogous operation generates the updated coarse‐scale features. The two resulting vectors are concatenated to form the final cellular embedding $Z_{\mathrm{fused}}\in R^{N\times 512}$.

### 5.4 Model Structure—Prototype Subspace Classifier

For the final classification stage, MethyAnno employs a prototype subspace classifier based on metric learning. Each cell type *c* is defined as a *k*‐dimensional subspace $S_{c}$ spanned by a set of learnable basis vectors that are initialized orthogonally and encouraged to remain close to orthonormality. To ensure numerical stability and geometric consistency, these basis vectors are initialized using an orthonormal initialization scheme. These vectors form the matrix $B_{c}\in R^{512\times k}$ (where *k*  =  8). For a given cellular embedding $Z_{\mathrm{fused}}\in R^{512}$, when $B_{c}^{T}\;B_{c}=I$, its orthogonal projection onto the subspace $S_{c}$ is given by $\mathrm{Pro}j_{S_{c}}\;\left(Z_{\mathrm{fused}}\right)=B_{c}\;B_{c}^{T}Z_{\mathrm{fused}}$. In MethyAnno, this condition is encouraged by the soft orthogonality regularization term L_ORTHO_, rather than strictly enforced during each forward propagation step. Therefore, this operation is used as an approximation of the orthogonal projection. Then, the projection error is calculated as the squared Euclidean distance between the original embedding and its approximation of projection:

$$d\;{\left(Z_{\mathrm{fused}},S_{c}\right)}^{2}={∥Z_{\mathrm{fused}}-\mathrm{Pro}j_{S_{c}}\left(Z_{\mathrm{fused}}\right)∥}_{2}^{2}\;,$$

where ${∥\cdot ∥}_{2}^{2}$ is the square of Euclidean distance.

The negative of this distance serves as the logit for class *c*. During inference, the cell is assigned to the cell type corresponding to the subspace that minimizes this projection error:

$$\;\hat{y}=\underset{c}{\mathrm{argmin}}d{\left(Z_{\mathrm{fused}},S_{c}\right)}^{2}$$

### 5.5 Model Structure—Identification of Novel Cell Types

To identify potential novel cell types, MethyAnno implemented a three‐stage pipeline comprising novelty scoring, adaptive thresholding, and density‐based clustering. First, we calculate a novelty score for each cell in the test set by quantifying its deviation from the training distribution. For a test embedding $z\in Z_{\mathrm{test}}$, the score is defined as the average Euclidean distance to its *k*‐nearest neighbors (*k*  =  10) within the training set **Z**_train_:

$\mathrm{Score}\;\left(z\right)=\frac{1}{k}\;\sum_{z_{\mathrm{train}}\in N_{k}\left(z,\;Z_{\mathrm{train}}\right)}∥z-z_{\mathrm{train}}{∥}_{2}$, where *N_k_* denotes the set of *k* nearest neighbors. Higher novelty scores indicate a greater epigenetic distance from known cell types.

Subsequently, to determine an adaptive threshold τ, we model the distribution of novelty scores *s* using a two‐component Gaussian mixture model. The probability density function is defined as:

$$P\;\left(s\right)=w_{1}N\left(s|\mu_{1},\sigma_{1}^{2}\right)+w_{2}N\left(s|\mu_{2},\sigma_{2}^{2}\right)$$

where μ_1_ < μ_2_, representing the distributions of known and putative novel populations, respectively, and N(*s*|μ, σ^2^) denotes the probability density function of a normal distribution with mean μ and variance σ^2^.

The decision threshold τ is identified as the intersection point of these two components. By equating the two weighted probability density functions, we derive the quadratic equation *As* ^2^ + *Bs* + *C*  =  0, with coefficients given by:

$$A=\frac{1}{2\sigma_{2}^{2}}\;-\frac{1}{2\sigma_{1}^{2}},\;B=\frac{\mu_{1}}{\sigma_{1}^{2}}\;-\frac{\mu_{2}}{\sigma_{2}^{2}},\;C=\frac{\mu_{2}^{2}}{2\sigma_{2}^{2}}\;-\frac{\mu_{1}^{2}}{2\sigma_{1}^{2}}+\ln\left(\frac{w_{1}\sigma_{2}}{w_{2}\sigma_{1}}\right)$$

The solution lying between μ_1_ and μ_2_ is selected as τ. Test cells with *s* > τ are designated as candidate novel cells for the subsequent clustering stage.

To distinguish genuine novel cell populations from random outliers, we apply a density‐based clustering strategy [51] to the candidate set $Z_{\mathrm{cand}}=\left\{z\in Z_{\mathrm{test}}|\mathrm{Score}\left(z\right)>\tau \right\}$. This approach posits that true novel types manifest as coherent, dense clusters in the embedding space. The clustering is parameterized by a neighborhood radius ε and a minimum sample count *m*. We set *m*  =  10 and align the radius with the novelty threshold (ε  =  τ), ensuring that the spatial scale for clustering is consistent with the novelty detection criterion. First, we define the ε‐neighborhood for each candidate **p** as $N_{\varepsilon }\;\left(p\right)=\left\{q\in Z_{\mathrm{cand}}|d\left(p,q\right)\leq \varepsilon \right\}$. A candidate is designated as a core cell if its neighborhood contains at least *m* cells. The set of core cells C is formally defined as: $C\;=\{p\in Z_{\mathrm{cand}}|\left|N_{\varepsilon }\left(p\right)\right|\geq m\}$. Clusters are formed by identifying connected components of core cells. Specifically, for an unvisited core cell p∈C, a new cluster *K_i_* is initialized and iteratively expanded to include all cells density‐reachable from **p**. This expansion recursively adds neighbors of any core cell added to the cluster until the density‐connected component is maximal. Candidates that are not core cells but fall within the ε‐neighborhood of a core sample are classified as border cells and assigned to the cluster. Any candidate neither core nor border is discarded as noise. The resulting sets $K_{1},\text{…},K_{M}$ represent the identified novel cell types.

### 5.6 Model Structure—Model Interpretation and Feature Contribution Analysis

To quantify feature contributions, we implement a gradient‐based attribution pipeline [52] that operates on the trained model parameters. In detail, for a target cell type *c*, we compute the global importance score of the *j*‐th feature by processing all *N* cells in the training set belonging to that cell type.

The process begins by defining a specific score function F_*c*_(**x**) to compute the gradients against. We utilize a contrastive logit score, calculated as the logit of the target class *c*, denoted as l_*c*_(**x**), minus the mean logit of all other classes, which centers the gradients on class‐discriminative signals:

$$F_{c}\;\left(x_{i}\right)=l_{c}\;\left(x_{i}\right)-\frac{1}{C-1}\sum_{t\neq c}l_{t}\left(x_{i}\right)$$

Next, we calculate the attribution **A**_*i*,*j*_ for each cell **x**_*i*_. This involves establishing a baseline input **x′** where both fine‐scale and coarse‐scale components are initialized as zero‐vectors. We then construct a linear interpolation path connecting this baseline **x′** to the actual input cell **x**_*i*_. This continuous path is discretized into *m* steps. For each step *k* (where k=1,…,m), we generate an intermediate input **x** ^(*k*)^ using the scaling factor α_*k*_ =  *k*/*m*. At each interpolated point, the gradients of the score function F_*c*_ with respect to the input features are computed via backpropagation. The attribution is derived by approximating the path integral: summing these gradients and scaling the result by the displacement from the baseline: 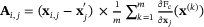, where $x^{\left(k\right)}=x^{\prime }+\frac{k}{m}\left(x_{i}-x^{\prime }\right)$ explicitly represents the *k*‐th interpolated cell along the trajectory. Finally, to obtain the feature importance representing the entire cell type, we aggregate the results across the dataset. The global importance score **A**_*j*_ for feature *j* is computed by averaging the individual attribution scores |**A**_*i*,*j*_| over all *N* cells belonging to type *c* in the training set. Specifically, in this study, the top 1000 features in fine‐scale representations with the highest **A**_*j*_ scores were identified as cell‐type‐specific signatures for downstream biological analysis, while background features are generated by excluding the union of all cell‐type‐specific features across all cell types from the whole‐genome feature set.

### 5.7 Composite Loss Function for Model Training

The training of the MethyAnno model is optimized using a composite loss function, which combines four distinct components designed to enforce specific properties on the learned representations and classifier structure. The total loss is formulated as a weighted sum:

$$\;L_{\mathrm{total}}=L_{\mathrm{CE}}+\lambda_{1}L_{\mathrm{SCL}}+\lambda_{2}L_{\mathrm{SUB}}+\lambda_{3}L_{\mathrm{ORTHO}}$$

where L_CE_ represents the cross‐entropy classification loss, L_SCL_ denotes the supervised contrastive loss applied to the projection heads, L_SUB_ is the subspace structuring loss designed to refine the geometric properties of class prototypes, and L_ORTHO_ imposes an orthogonality constraint on the basis vectors. The hyperparameters λ_*i*_ control the contribution of each regularization loss. Specifically, we empirically set these weights to λ_1_ =  0.8, λ_2_ =  0.1, and λ_3_ =  0.1 to effectively balance the regularization terms during the training process. A comprehensive list of all hyperparameter configurations is provided in Table S2. Each component is detailed below.

First, to ensure accurate cell type classification, we employ the standard multi‐class cross‐entropy loss. The predicted class probabilities are obtained by applying the softmax function to the subspace‐based logits derived previously. The loss function is defined as:

$$\;L_{\mathrm{CE}}=-\frac{1}{N}\sum_{i=1}^{N}\sum_{c=1}^{C}y_{i,c}\log\left(p_{i,c}\right)$$

where *N* is the batch size, *C* is the total number of cell types, *y*_*i*,*c*_ is the binary indicator (0 or 1) for the true label, and *p*_*i*,*c*_ is the predicted probability that cell *i* belongs to class *c*.

Second, to enforce intra‐class compactness and inter‐class separability, we apply the supervised contrastive loss (L_SCL_) to the L2‐normalized outputs from the projection heads. Let ${\left\{{z^{\prime }}_{i}\right\}}_{i=1}^{2N}$ denote the combined batch of feature embeddings after projection from both branches. The loss for an anchor cell **z′**_*i*_ is defined as:

$${\;\;L}_{\mathrm{SCL}}^{i}=-\;\frac{1}{\left|P\left(i\right)\right|}\sum_{p\in P\left(i\right)}\log\frac{\exp\left(\mathrm{sim}\left({z^{\prime }}_{i},{z^{\prime }}_{p}\right)/\tau_{1}\right)}{\sum_{a\in A\left(i\right)}\exp\left(\mathrm{sim}\left({z^{\prime }}_{i},{z^{\prime }}_{a}\right)/\tau_{1}\right)}$$

where *A*(*i*) represents the set of all indices in the batch excluding *i*, and *P*(*i*) denotes the set of indices for positive samples, including *i* (i.e., those sharing the same cell type as *i*). The term sim(**u**, **v**) denotes cosine similarity, and τ_1_ is the temperature scalar. The final L_SCL_ is computed by averaging $L_{\mathrm{SCL}}^{i}$ over the entire batch.

Third, to ensure that the learnable class subspaces are well‐structured and maximally separated, we introduce a combined subspace loss. This loss itself is a weighted combination of two contrastive terms, dynamically controlled by a linearly scheduled parameter α_*t*_ that decreases over training epochs *t*:

$$\;L_{\mathrm{SUB}}=\alpha_{t}L_{\mathrm{all}}+\left(1-\alpha_{t}\right)L_{\mathrm{center}}$$

where α_*t*_ is defined as $\alpha_{t}=1-\frac{t}{T_{\mathrm{total}}}$, and *T*_total_ represents the total number of training epochs.

Importantly, instead of enforcing intra‐class compactness via contrastive learning, we rely on the orthogonality constraint (L_ORTHO_) to structure the internal geometry of each subspace. Consequently, the contrastive objectives in L_SUB_ are utilized purely for inter‐vector and inter‐class separation. The two loss terms operate at different structural levels to achieve this.

The first term, L_all_, operates at the fine‐grained level. Its role is to enforce instance‐level discrimination and promote global diversity among the basis vectors. Instead of strict pair‐wise masking, L_all_ treats the entire set of basis vectors as a global negative pool. For its calculation, it evaluates the exponentiated cosine similarity between a given basis vector and all basis vectors across the entire subspace manifold. Let *b*_*c*,*i*_ denote the *i*‐th basis vector of class *c*, *k* be the number of basis vectors per subspace, and τ_2_ be the temperature parameter. The term sim(*u*, *v*) denotes cosine similarity. The calculation is formulated as:

$$\;L_{\mathrm{all}}=-\;\frac{1}{C\cdot k}\sum_{c=1}^{C}\sum_{i=1}^{k}\log\frac{\exp\left(\mathrm{sim}\left(b_{c,i},b_{c,i}\right)/\tau_{2}\right)}{\sum_{\;c^{\prime }=1}^{C}\sum_{j=1}^{k}\exp\left(\mathrm{sim}\left(b_{c,i},b_{c^{\prime },j}\right)/\tau_{2}\right)}$$

The second term, L_center_, operates at the macroscopic class level. Its primary role is to explicitly enforce inter‐class separability at the subspace level, ensuring that different class subspaces are well separated in the embedding space. This term is calculated based on the mean representation (center) of each class subspace, denoted as μ_*c*_. It operates by encouraging each class center to be maximally similar to itself while simultaneously minimizing its similarity to all other distinct class centers:

$$\;L_{\mathrm{center}}=-\frac{1}{C}\sum_{c=1}^{C}\log\frac{\exp\left(\mathrm{sim}\left(\mu_{c},\mu_{c}\right)/\tau_{2}\right)}{\sum_{j=1}^{C}\exp\left(\mathrm{sim}\left(\mu_{c},\mu_{j}\right)/\tau_{2}\right)}$$

Finally, to encourage linear independence and improve the geometric stability of the learned subspaces, we impose an orthogonality regularization term on the basis vectors. This regularization encourages orthonormality by minimizing the deviation of the Gram matrix $B_{c}^{T}B_{c}$ from the identity matrix **I**. For each cell type subspace **B**_*c*_, the loss is defined as:

$$\;L_{\mathrm{ORTHO}}^{c}={∥B_{c}^{T}B_{c}-I∥}_{F}^{2}$$

where || · ||_F_ is the Frobenius norm. The final L_ORTHO_ is the average over all class subspaces.

### 5.8 Data Collection and Preprocessing

In this study, we collected scDNAm datasets spanning different species, tissues, and sequencing technologies. For the mouse brain, we obtained snmC‐seq2 datasets covering six anatomical regions [4, 8]. Specifically, we collected paired datasets from two distinct donors for the caudoputamen (CP), primary motor area (MOp), secondary motor area (MOs), auditory area (AUD), and visual area (VIS), along with a single dataset for the retrosplenial area (RSP). For the human brain, we acquired data generated via two distinct sequencing protocols [9]. We collected snmC‐seq3 data from five cortical regions, each represented by two donors: the middle frontal gyrus (A46), supraparietal lobule—posterosuperior parietal cortex (A5‐A7), anterior cingulate cortex (ACC), primary motor cortex (M1C), and middle temporal gyrus (MTG). Additionally, we included two snm3C‐seq datasets for the M1C and MTG regions. Finally, to extend tissue diversity, we collected five human adipose tissue samples sequenced using snm3C‐seq3 [25]. Detailed statistical information regarding all data utilized in this study is provided in Table S3, and detailed descriptions of all cell types mentioned in this study are provided in Table S6.

For the human and mouse brain datasets, where raw sequencing data were available, we constructed the input cell‐by‐region matrix $X\in R^{N\times R_{0}}$ by aggregating CpG sites into 10‐kb genomic bins, consistent with the resolution adopted in a recent scDNAm study [4]. In this matrix, each entry *x_ij_* in the matrix represents the average methylation level in genomic region *j* for cell *i*. In contrast, for the Human‐Adipose dataset, since raw sequencing data were not accessible, we directly utilized the processed matrix at 5‐kb resolution provided by the original study. Subsequently, genomic regions with missing values across all cells were removed, and all remaining missing values were imputed using the feature‐wise median. Next, a multi‐scale information representation was generated from the imputed matrix, now referred to as the fine‐scale matrix $X_{\mathrm{fine}}\in R^{N\times R_{1}}$. A coarse‐scale matrix, $X_{\mathrm{coarse}}\in R^{N\times R_{2}}$, with a 10‐fold lower resolution was created by averaging every *k*  =  10 adjacent fine‐scale bins within each chromosome. The value of an entry $x_{ir}^{\mathrm{coarse}}$ in the coarse‐scale matrix is:

$$\;x_{ir}^{\mathrm{coarse}}=\frac{1}{k}\;\sum_{j=kr+1}^{k\left(r+1\right)}x_{ij}^{\mathrm{fine}}$$

This averaging was performed exclusively on non‐missing values. Any new missing values that arose from windows containing only empty bins were then imputed using the same feature‐wise median strategy [53].

Finally, feature selection was performed using an F‐test (one‐way Analysis of Variance, ANOVA). To ensure the test set remained unseen during this process, the F‐statistic was calculated exclusively on the training data. The one‐way ANOVA evaluates the null hypothesis that the population means of a specific feature are equal across all defined cell types. For each genomic region, the F‐statistic quantifies the ratio of the variance between different cell types to the variance within the same cell types. Mathematically, let *C* be the total number of cell types, *N* be the total number of cells in the training set, *n_i_* be the number of cells in cell type *i*, *x_ij_* be the feature value for cell *j* in cell type *i*, ${\bar{x}}_{i}$ be the mean feature value within cell type *i*, and $\bar{x}$ be the overall mean of the feature across all cells. The F‐statistic is calculated as:

$$F=\frac{MSB}{MSW}$$

where *MSB* (Mean Square Between) represents the between‐group variability, calculated as:

$$MSB=\frac{\sum_{i=1}^{C}n_{i}{\left({\bar{x}}_{i}-\bar{x}\right)}^{2}}{C-1}$$

and *MSW* (Mean Square Within) represents the within‐group variability, calculated as:

$$MSW=\frac{\sum_{i=1}^{C}\sum_{j=1}^{n_{i}}{\left(x_{ij}-{\bar{x}}_{i}\right)}^{2}}{N-C}$$

A higher F‐statistic indicates that the corresponding genomic region exhibits significant heterogeneity across different cell types while maintaining high stability within the same cell type. During the calculation, we mathematically handled numerical singularities by replacing undefined values with zeros. Subsequently, we identified the top features based on their statistical significance. This selection resulted in final feature dimensions of *D*_1_ =  5000 for the fine‐scale data and *D*_2_ =  3000 for the coarse‐scale data. This identical set of selected features was then used to filter both the training and test sets, yielding the final multi‐scale information for all downstream model training and evaluation.

### 5.9 Baseline Methods

Given the current absence of automated cell type annotation tools specifically tailored for scDNAm data, we benchmarked MethyAnno against representative methods that have demonstrated strong performance in related fields. In this study, we benchmarked MethyAnno against six baseline methods, including four conventional machine learning methods and two variations of MINGLE [12], a state‐of‐the‐art annotation method designed for scATAC‐seq data. The four conventional machine learning methods include support vector machine (SVM), random forest (RF), eXtreme Gradient Boosting (XGBoost), and K nearest neighbors (KNN), which were recommended by recent benchmark studies [11, 26]. For conventional machine learning baselines, we evaluated both default‐parameter and hyperparameter‐tuned settings. The corresponding results obtained using default‐parameter baselines are provided in Figures S4–S7. Additionally, to assess the model performance of identifying novel cell types, we compared with SVM with rejection option (SVMrejection), which has been confirmed the ability to accurately identify novel cell types in the recent benchmark study [26]. Furthermore, we compared MethyAnno against MINGLE, a state‐of‐the‐art annotation framework designed for scATAC‐seq data. As the direct application of MINGLE to raw scDNAm data yielded poor performance, we applied two distinct preprocessing strategies to adapt the data for this method, resulting in two variants: MINGLE_TF and MINGLE_No_TF. Comprehensive implementation details for all these baseline methods are provided in Text S2.

## Author Contributions

**Yuhang Jia**: methodology, investigation, validation, formal analysis, writing – original draft. **Siyu Li**: validation, writing – review and editing, data curation, methodology. **Songming Tang**: validation. **Keju Gu**: validation. **Shengquan Chen**: conceptualization, supervision, funding acquisition, writing – review and editing.

## Conflicts of Interest

The authors declare no conflicts of interest.

## Supporting information




**Supporting File**: advs77524‐sup‐0001‐SuppMat.pdf

## Data Availability

All datasets used in this study are publicly available from the National Center for Biotechnology Information (NCBI) Gene Expression Omnibus (GEO). For the human cerebral cortex datasets, the corresponding accession number is GSE215353. Detailed information and associated accession numbers regarding all specific brain regions used in this study are provided in Table S3. The mouse brain datasets can be derived from GSE132489 and GSE213262, detailed accession numbers for each subdataset are also summarized in the Table S3. Moreover, the accession number of the human adipose dataset is GSE297262. The source codes of MethyAnno are publicly available in the GitHub repository at https://github.com/BioX‐NKU/MethyAnno under an MIT license. We have released MethyAnno as a python package for easy installation, and provided detailed tutorials on GitHub.
