# Supplementary material for: MethyAnno: An Interpretable Automated Annotation Method Leveraging Multi‐Scale Information and Metric Learning Framework for scDNAm Data
Source: Adv Sci (Weinh). 2026 Sep 1:e77524. Online ahead of print. doi: 10.1002/advs.77524 (PMC13534786; doi:10.1002/advs.77524)
Supplement: Supplementary file 1 [file ADVS-9999-e77524-s001.pdf]

Supporting Information

MethyAnno: An Interpretable Automated Annotation Method Leveraging Multi-scale Information and Metric Learning Framework for scDNAm Data

*Yuhang Jia, Siyu Li, Songming Tang, Keju Gu, Shengquan Chen**

Contents

Supplementary Texts **3**

Text S1 3

Text S2 8

Text S3 11

Text S4 14

Supplementary Figures **15**

Figure S1 15

Figure S2 16

Figure S3 17

Figure S4 18

Figure S5 19

Figure S6 20

Figure S7 21

Supplementary Tables **22**

Table S1 22

Table S2 23

Table S3 24

Table S4 25

Table S5 26

Table S6 27

References **28**

Supplementary Texts

Text S1: Implementation details of ablation studies

To ensure the comprehensiveness and robustness of our ablation experiments, we utilized datasets from the three major tissues: the human adipose, the mouse brain, and the human brain. For each tissue type, we rigorously paired two datasets derived from the same anatomical region but from different donors to serve as the training and testing sets.

To systematically evaluate the contribution of each component in MethyAnno, we performed ablation experiments using 10 independent random seeds and reported the mean ± standard deviation across runs. The evaluated variants included removing the multi-scale input branch, removing the cross-attention module, removing the supervised contrastive loss, removing the subspace structuring loss, removing the orthogonality constraint, and replacing the prototype subspace with a single-vector representation. Because scDNAm cell type annotation is often affected by substantial class imbalance and the presence of rare cell types, we consider Macro-F1 particularly informative for evaluating balanced classification performance. Unlike Acc, which can be dominated by abundant cell types, Macro-F1 assigns equal weight to each class and is therefore more sensitive to performance changes in underrepresented cell populations. Therefore, in the following interpretation, we focus mainly on Macro-F1 while also reporting Acc and Kappa for completeness. The detailed results are provided in Supplementary Table S1.

Among all ablation variants, the single-branch variant, which used only the original data matrix constructed with a 10-kb window length as input, showed the most consistent decrease in Macro-F1. Compared with the full MethyAnno model, Macro-F1 decreased from 0.9522 ± 0.0058 to 0.9447 ± 0.0071 on the human adipose tissue, from 0.8206 ± 0.0367 to 0.7872 ± 0.0295 on the mouse brain tissue, and from 0.9818 ± 0.0062 to 0.9346 ± 0.0126 on the human brain tissue. Although the changes in Acc and Kappa were relatively small, the consistent decrease in Macro-F1 indicates that removing the multi-scale input weakens balanced classification performance. These

results suggest that multi-scale methylation features provide complementary information beyond the original 10-kb resolution and help MethyAnno capture both local methylation patterns and broader regional epigenetic signals.

The no cross-attention variant was used to evaluate the effect of adaptive feature fusion between the two scale-specific branches. In this variant, the features extracted from the two branches were directly concatenated before being fed into the prototype classifier. Removing cross-attention resulted in more moderate and dataset-dependent effects than removing the multi-scale branch. On the human adipose tissue dataset, Macro-F1 decreased from 0.9522 ± 0.0058 to 0.9456 ± 0.0131. On the human brain tissue dataset, Macro-F1 decreased from 0.9818 ± 0.0062 to 0.9726 ± 0.0168. On the mouse brain tissue dataset, however, the no cross-attention variant achieved a similar Macro-F1 to the full model, with a slight increase from 0.8206 ± 0.0367 to 0.8225 ± 0.0507. These results indicate that the contribution of cross-attention is not uniform across tissues. Cross-attention may help integrate scale-specific methylation features by enabling adaptive information exchange between the two branches, but its empirical benefit depends on the degree of complementarity between fine-scale and coarse-scale methylation features in each dataset.

The effects of the loss-related components were also examined. Among these loss-related variants, removing the supervised contrastive loss showed the clearest adverse effect on balanced classification performance across the evaluated datasets, indicating that this loss helps shape discriminative cell embeddings and promotes better separation among cell types. In contrast, the effects of the two subspace-related regularization terms were more dataset-dependent. The subspace structuring loss encourages cell embeddings to align with their corresponding class-specific prototype subspaces, thereby strengthening the relationship between learned cell representations and the prototype-based classifier. When this loss was removed, Macro-F1 changed from 0.9522 ± 0.0058 to 0.9494 ± 0.0124 on the human adipose tissue dataset, from 0.8206 ± 0.0367 to 0.8124 ± 0.0464 on the mouse brain tissue dataset, and from 0.9818 ± 0.0062 to 0.9837 ± 0.0023 on the human brain tissue dataset. Thus, removing the subspace structuring loss reduced Macro-F1 in the human adipose and mouse brain

datasets, whereas its effect was negligible or slightly positive in the human brain dataset. The orthogonality constraint mainly acts on the geometry of the prototype subspaces by encouraging the basis vectors to remain less redundant and more structurally organized. Removing this constraint changed Macro-F1 from 0.9522 ± 0.0058 to 0.9492 ± 0.0070 on the human adipose tissue dataset, from 0.8206 ± 0.0367 to 0.8227 ± 0.0417 on the mouse brain tissue dataset, and from 0.9818 ± 0.0062 to 0.9774 ± 0.0158 on the human brain tissue dataset. These results suggest that the orthogonality constraint can help preserve the geometric structure of the prototype subspaces, but its effect on global classification metrics is also dataset-dependent.

Although the empirical gains brought by these loss-related components were not always pronounced in global metrics such as Acc and Kappa, their effects on Macro-F1 indicate that they can contribute to balanced classification performance in a dataset-dependent manner. More importantly, these losses play complementary roles in the subspace-based metric learning formulation of MethyAnno. The supervised contrastive loss shapes the global discriminative structure of the embedding space, the subspace structuring loss links cell-level representations with class-specific prototype subspaces, and the orthogonality constraint preserves the geometric quality of the learned subspaces. Therefore, these components are better interpreted as theoretically motivated auxiliary objectives that support discriminative representation learning, prototype-subspace alignment, and structured subspace geometry, rather than as modules that uniformly improve every metric across all datasets.

The single-vector variant was further evaluated to assess the contribution of representing each cell type with a prototype subspace rather than a single representative vector. The single-vector variant achieved performance comparable to the full prototype-subspace model in several settings. On the human adipose tissue dataset, the full MethyAnno model achieved a Macro-F1 of 0.9522 ± 0.0058, while the single-vector variant achieved a slightly higher but comparable Macro-F1 of 0.9531 ± 0.0096. On the mouse brain tissue dataset, the single-vector variant achieved a Macro-F1 of 0.8378 ± 0.0494, which was higher than that of the full MethyAnno model (0.8206 ± 0.0367). On the human brain tissue dataset, the full prototype-subspace model achieved

a Macro-F1 of 0.9818 ± 0.0062, whereas the single-vector variant achieved 0.9806 ± 0.0079.

Although the current ablation results do not show that prototype subspaces uniformly outperform the single-vector variant across all datasets and metrics, prototype subspaces still have several theoretical advantages over a single-vector representation. First, a single vector can be regarded as a special case of the prototype-subspace formulation when the subspace dimension is reduced to one; therefore, prototype subspaces provide a more general and flexible class representation. Second, unlike a single-vector prototype, which represents each cell type by one representative center or direction, a prototype subspace can, in principle, characterize each cell type using multiple basis directions. This is particularly relevant for scDNAm data, where cells with the same annotation may still exhibit substantial intra-class epigenetic heterogeneity, donor-related variation, or latent cell-state differences. Third, prototype subspaces avoid forcing all cells from the same class to collapse around a single point and instead allow class-specific embeddings to vary within a structured low-dimensional space. This makes the representation more suitable for cell types with complex or elongated distributions in the embedding space. Finally, prototype subspaces are more consistent with the intended subspace-based metric learning formulation of MethyAnno, because class identity is defined by alignment with a class-specific subspace rather than similarity to a single prototype vector. Therefore, while the empirical benefit of prototype subspaces is dataset-dependent, they remain a theoretically motivated and more expressive representation mechanism for modeling intra-class epigenetic heterogeneity.

Overall, the multi-seed ablation analysis provides a more balanced understanding of the contribution of each component in MethyAnno. The multi-scale input branch showed the clearest and most consistent empirical contribution, particularly in Macro-F1, whereas cross-attention, the loss-related objectives, and the prototype-subspace classifier showed more dataset-dependent effects. These findings suggest that the components of MethyAnno contribute in complementary ways: the multi-scale branch enriches methylation feature representation, cross-attention supports adaptive feature

fusion across genomic resolutions, the loss-related objectives regularize discriminative and geometrically structured representation learning, and the prototype subspace provides a more expressive formulation for modeling intra-class epigenetic heterogeneity.

Text S2: Implementation details of baseline methods

We evaluated the conventional machine learning baselines under two settings. The first setting used the default parameter settings, including the default parameters of SVM, RF, and XGBoost, as well as the KNN9 and KNN50. The second setting used hyperparameter-tuned versions of SVM, RF, KNN, and XGBoost. The main text reports the results obtained from the tuned baseline models, because this provides a fairer comparison with MethyAnno. The results obtained using the default parameter settings are provided in the Supplementary Materials for transparency.

For hyperparameter tuning, we performed five-fold cross-validation with a fixed random seed. For each conventional machine learning baseline, grid search was conducted over key parameters according to the corresponding method documentation. Macro-F1 was used as the primary metric for selecting the final parameter combination, because it is more suitable for imbalanced cell type annotation and rare cell type recognition. Accuracy and Kappa were also recorded as complementary evaluation metrics. After five-fold cross-validation, the average Macro-F1 score was calculated for each parameter combination. If multiple parameter combinations achieved the same best Macro-F1 score, these combinations were first sorted by parameter order, and one combination was selected using the fixed random seed 123. The selected tuned parameters were then fixed and applied to all benchmark experiments. The parameter names, parameter descriptions, searched value ranges, and selected tuned parameter settings are summarized in Table S4.

MINGLE is a mutual information-based interpretable framework that leverages cellular similarities and topological structures to accurately annotate scATAC-seq data and identify novel cell types via a convex hull-based strategy. Additionally, since the direct application of MINGLE to raw scDNAm data yielded poor performance, we implemented two distinct preprocessing strategies. The first variant, designated as MINGLE_TF, involves subtracting the methylation values from one to obtain non-methylated scores, followed by TF-IDF normalization. The second variant, designated as MINGLE_No_TF, involves the same subtraction step but excludes any additional

normalization. For all other implementation details, we adhered to the official tutorial provided on GitHub (https://github.com/BioX-NKU/MINGLE) and executed the framework with default parameters. Notably, due to occasional runtime errors encountered by the MINGLE framework during novel cell type identification, we exclusively presented results from successful executions in this study.

Support vector machine (SVM) is a supervised learning algorithm designed to identify the optimal hyperplane that separates data into distinct categories. In the default parameter setting, we implemented this method using the sklearn.svm module from the scikit-learn library and utilized the default settings for our experiments. In the tuned setting, we optimized the regularization parameter C, kernel function, and polynomial degree. The final selected SVM parameters were C=1.5 and kernel=linear. Since the selected kernel was linear, the polynomial degree parameter was not applicable in the final model.

Random forest (RF) is an ensemble learning technique that builds numerous decision trees during the training phase and determines the final classification based on the mode of the outputs from individual trees. In the default parameter setting, we conducted our experiments using the RandomForestClassifier class from the sklearn.ensemble module in scikit-learn with default settings. In the tuned setting, we optimized the number of trees, splitting criterion, and maximum tree depth. The final selected RF parameters were n_estimators=500, criterion=entropy, and max_depth=220.

K-nearest neighbors (KNN) is an instance-based algorithm that assigns a class label to a data point based on the majority vote of its nearest neighbors in the feature space. In the default parameter setting, we utilized the KNeighborsClassifier class from the sklearn.neighbors module in scikit-learn. Following the recommendations of a recent review[1], two configurations were implemented: KNN9 and KNN50, where the n_neighbors parameter was set to 9 and 50, respectively. In the tuned setting, we optimized the number of neighbors, the neighbor weighting function, and the neighbor search algorithm. The final selected KNN parameters were n_neighbors=5, weights=distance, and algorithm=kd_tree.

Extreme gradient boosting (XGBoost) is an optimized distributed gradient boosting library designed for efficiency and scalability. It operates by iteratively training an ensemble of decision trees to minimize a regularized objective function, effectively capturing complex data patterns. In the default parameter setting, we implemented XGBoost using the XGBClassifier interface from the xgboost Python package and applied the default parameter settings for the classification tasks. In the tuned setting, we optimized the number of boosting rounds, maximum tree depth, and maximum number of leaves. The final selected XGBoost parameters were n_estimators=480, max_depth=160, and max_leaves=40.

SVMrejection extends the standard SVM framework by incorporating a mechanism to detect and exclude ambiguous or uncertain classifications. This approach uses posterior probabilities to assess prediction confidence. Consistent with recommendations from a recent benchmark study, we applied a probability threshold of 0.7 to designate cells as “Novel”[2].

Text S3: Details of model evaluation metrics

To comprehensively evaluate the performance of MethyAnno and other baseline methods, we employed distinct sets of metrics tailored to the specific nature of the classification task. For multi-class annotation tasks, we assessed the model using Accuracy (Acc), Cohen's Kappa value (Kappa), and the macro F1 score (Macro-F1). These metrics provide a holistic view of classification performance across all cell types. For binary classification tasks, we utilized Precision, Recall, and the binary F1-score to measure the specific detection capability. Furthermore, in the experiments involving different sequencing technologies, in addition to the Kappa score used for multi-class evaluation, we incorporated the Adjusted Rand Index (ARI) to specifically measure the consistency between two sets of prediction results.

In multi-class annotation experiments where all test set cell types were present in the training set, we evaluated performance using three standard metrics as recommended by recent benchmark studies[1,2].

Accuracy (Acc): The proportion of correctly classified cells. For a set of N cells, with true labels y_i and predicted labels $\hat{y}_i$, accuracy is defined as:

$$\mathrm{Acc} = \frac{1}{N}\sum_{i=1}^{N} \mathbb{I}(y_i = \hat{y}_i)$$

where $\mathbb{I}$ is the indicator function.

Cohen's Kappa value (Kappa): A metric designed to measure the agreement between two classification results while accounting for the possibility of chance agreement. In the context of multi-class cell type prediction, it assesses the concordance between the predicted labels and the ground truth labels. Additionally, within the cross-technique experiments, it is utilized to evaluate the consistency between two distinct sets of prediction results. It is calculated as:

$$\mathrm{Kappa} = \frac{p_{\mathrm{o}} - p_{\mathrm{e}}}{1 - p_{\mathrm{e}}}$$

where p_{o} is the observed agreement (Accuracy) and p_{e} is the expected agreement by chance.

Macro F1-Score (Macro-F1): The Macro-F1 score is defined as the unweighted

arithmetic mean of the F1-scores calculated for each individual class. This metric is particularly suitable for multi-class classification problems because it treats all classes equally regardless of their sample size. Consequently, it remains robust against class imbalance and provides a comprehensive evaluation across all cell types. Specifically, it is computed by first determining the F1-score (the harmonic mean of precision and recall) for each class independently, and then taking the simple average of these per-class F1-scores.

For experiments involving the identification of novel cell types and other binary classification problems, the performance was assessed by Precision, Recall and binary F1-score.

Precision: Precision quantifies the proportion of true positive instances relative to the total number of positive predictions. It serves as a metric to evaluate the reliability of the model when predicting the positive class.

$$\mathrm{Precision} = \frac{\mathrm{TP}}{\mathrm{TP} + \mathrm{FP}}$$

where TP and FP represent the counts of True Positives and False Positives respectively.

Recall: Recall, also known as sensitivity or the true positive rate, quantifies the proportion of actual positive instances that are correctly identified by the model. It serves as a measure of the model's effectiveness in capturing all relevant positive samples.

$$\mathrm{Recall} = \frac{\mathrm{TP}}{\mathrm{TP} + \mathrm{FN}}$$

where TP, FP, and FN represent the counts of True Positives, False Positives, and False Negatives, respectively.

F1-score: The F1-score serves as a comprehensive metric for evaluating binary classification models. Defined as the harmonic mean of precision and recall, it offers a unified measure that effectively balances the trade-off between prediction exactness and sensitivity.

$$\mathrm{F1-score} = \frac{2 * \mathrm{Precision} * \mathrm{Recall}}{\mathrm{Precision} + \mathrm{Recall}}$$

In the cross-technique experiments, in addition to the Cohen’s Kappa utilized for

multi-class evaluation, we employed the Adjusted Rand Index (ARI) to specifically assess the consistency of prediction results generated by models trained on datasets from different sequencing technologies.

ARI quantifies the similarity between two data partitions (here, the two sets of predicted labels) while correcting for the probability of agreement occurring by chance. It ranges from -1 to 1, where a score of 1 indicates perfect concordance and 0 indicates random agreement. The metric is calculated as follows:

$$\mathrm{ARI} = \frac{\sum_{ij}\binom{n_{ij}}{2} - \left[\sum_i\binom{a_i}{2}\sum_j\binom{b_j}{2}\right]/\binom{n}{2}}{\frac{1}{2}\left[\sum_i\binom{a_i}{2} + \sum_j\binom{b_j}{2}\right] - \left[\sum_i\binom{a_i}{2}\sum_j\binom{b_j}{2}\right]/\binom{n}{2}}$$

where n_{ij} is the number of objects common to classes i and j in the contingency table, a_i is the sum of the i-th row, b_j is the sum of the j-th column, and n is the total number of samples.

Furthermore, when testing the robustness of the method by varying the degree of imbalance of the dataset, we quantify the imbalance degree using the normalized entropy of the cell type size distribution as follows:

$$\mathrm{I} = 1 + \frac{1}{\log C}\sum_{c=1}^{C}\frac{n_c}{N}\log\frac{n_c}{N}$$

where C denotes the number of cell types in the dataset, n_c denotes the number of cells in the c-th cell type, N denotes the total number of cells in the dataset.

Specifically, this imbalance degree takes a value of 1 if a single cell type comprises all the cells in the dataset, and a value of 0 if all cell types have an identical number of cells. Additionally, the proportion of the major cell type refers to the ratio of the cell type with the highest cell counts to the total number of cells in the dataset.

Text S4: Implementation details of downstream analyses

Partitioned linkage disequilibrium score regression (LDSC) is a statistical framework designed to estimate the proportion of trait heritability attributable to specific genomic annotations[3]. Originally developed to refine the interpretation of genome-wide association studies (GWAS), LDSC utilizes linkage disequilibrium to disentangle true polygenic signals from confounding factors such as population stratification and cryptic relatedness. In this study, we employed partitioned LDSC with standard parameters to assess the heritability enrichment of brain-related phenotypes within the specific features defined for each cell type.

SNPsea is a computational algorithm developed to link genetic risk loci with the specific tissues, cell types, and biological pathways they influence[4]. By analyzing single-nucleotide polymorphisms (SNPs), the tool evaluates whether trait-associated genomic loci exhibit specificity to particular biological conditions. We utilized SNPsea with default parameters to quantify the enrichment of our cell-type-specific features against expression profiles across 79 distinct tissues. The results for the top 30 tissues showing statistically significant enrichment are presented.

The gene regulatory enrichment analysis tool (GREAT) is a bioinformatics resource used to associate genomic intervals with functional biological pathways and regulatory roles[5]. To gain functional insights into specific cell subpopulations, we analyzed the cell-type-specific features identified by our method using the GREAT server with default parameters. This analysis highlighted significant biological processes associated with these regions, and the most significant processes are displayed in the Figure 3C and Figure 4C.

Supplementary Figures

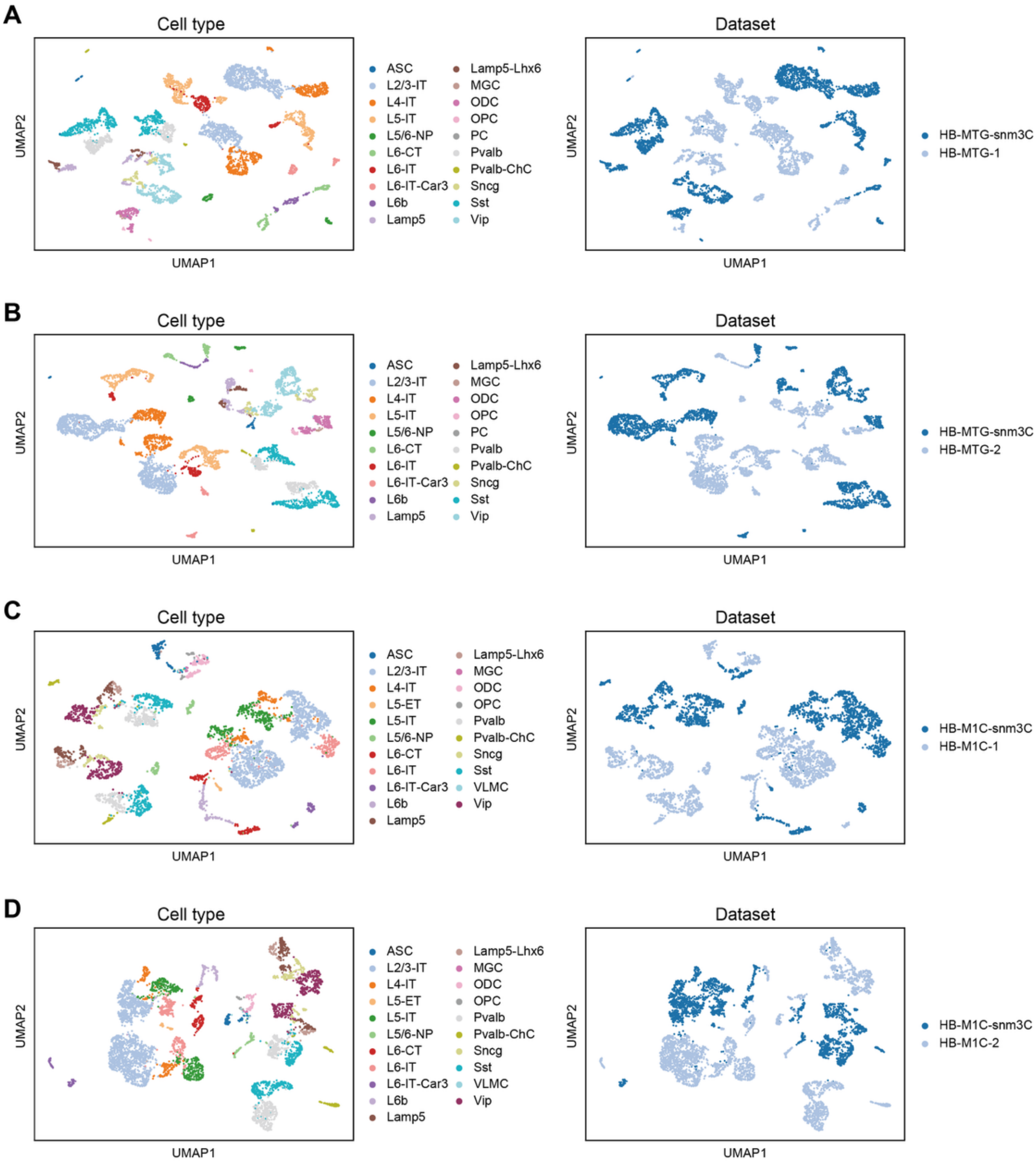

Figure S1. UMAP visualization of scDNAm datasets from the same anatomical regions sequenced by different technologies (snmC-seq3 and snm3C-seq). In each row, cells are colored by cell type and dataset source. **A, B**. Middle Temporal Gyrus (MTG) region. The snm3C-seq dataset is concatenated with (**A**) the HB-MTG-1 dataset and (**B**) the HB-MTG-2 dataset. **C, D**. Primary Motor Cortex (M1C) region. The snm3C-seq dataset is concatenated with (**C**) the HB-M1C-1 dataset and (**D**) the HB-M1C-2 dataset.

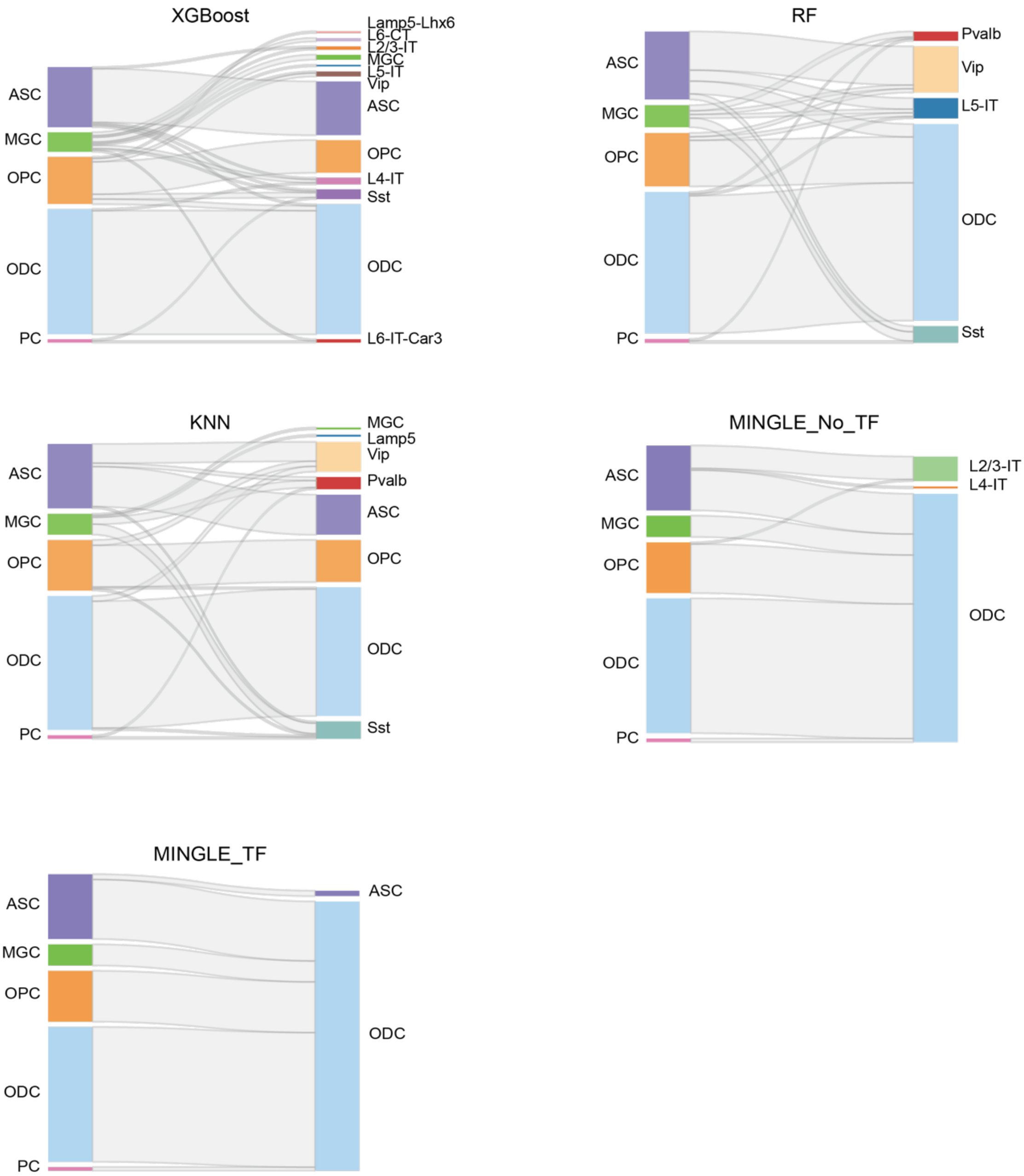

Figure S2. The annotation results of baseline methods (tuned XGBoost, tuned RF, tuned KNN, MINGLE_No_TF, MINGLE_TF) for non-neuronal cell types in the HB-MTG-2 dataset, presented in Sankey diagrams.

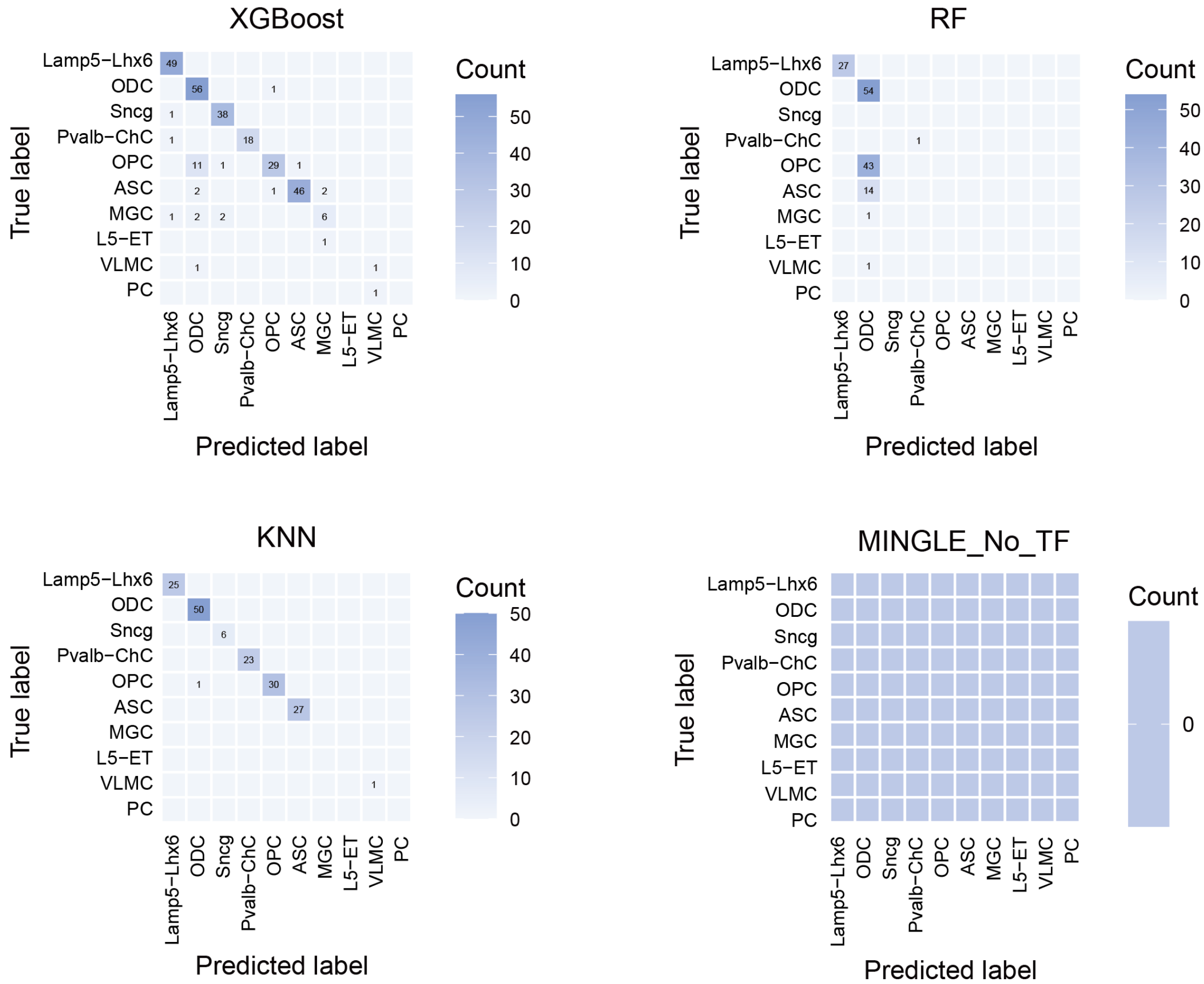

Figure S3. Prediction results of baseline methods (tuned XGBoost, tuned RF, tuned KNN, MINGLE_No_TF), which were trained on the HB-MTG-1 dataset and tested on the HB-ACC-1 dataset. Only cell types with fewer than 50 training cells are visualized, where each value represents the number of cells from the column cell type predicted as the corresponding row cell type.

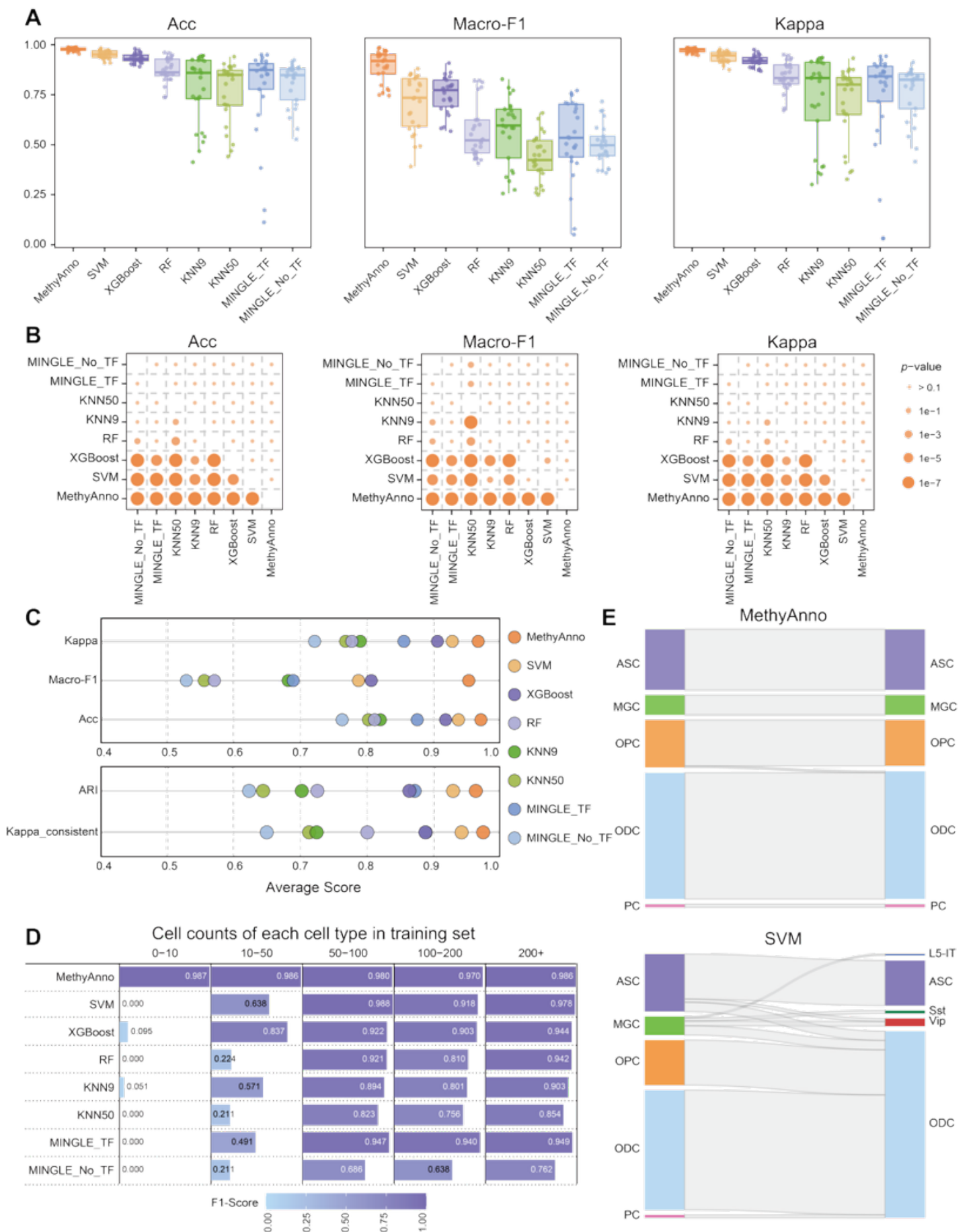

Figure S4. Performance of MethyAnno and baseline methods in cross-donor and cross-sequencing technology scenarios, where all machine learning baselines were implemented with their default parameter configurations. **A.** The performance of MethyAnno and baseline methods across 25 intra-region cross-donor experiments, presented in box plots. The scatter points represent results of each experimental run. **B.** Heatmap of *p*-values from one-sided paired Wilcoxon signed-rank tests determining whether the method in the column achieved significantly higher metrics than the method in the row. **C.** The average multi-class classification metrics of MethyAnno and baseline methods in cross-sequencing technology experiments. **D.** The average consistency metrics of MethyAnno and baseline methods in cross-sequencing technology experiments. **E.** The average F1-scores of MethyAnno and baseline methods for each cell type stratified by the cell counts in the training set. **F.** The annotation results of MethyAnno and SVM for non-neuronal cell types in the HB-MTG-2 dataset, presented in Sankey diagrams.

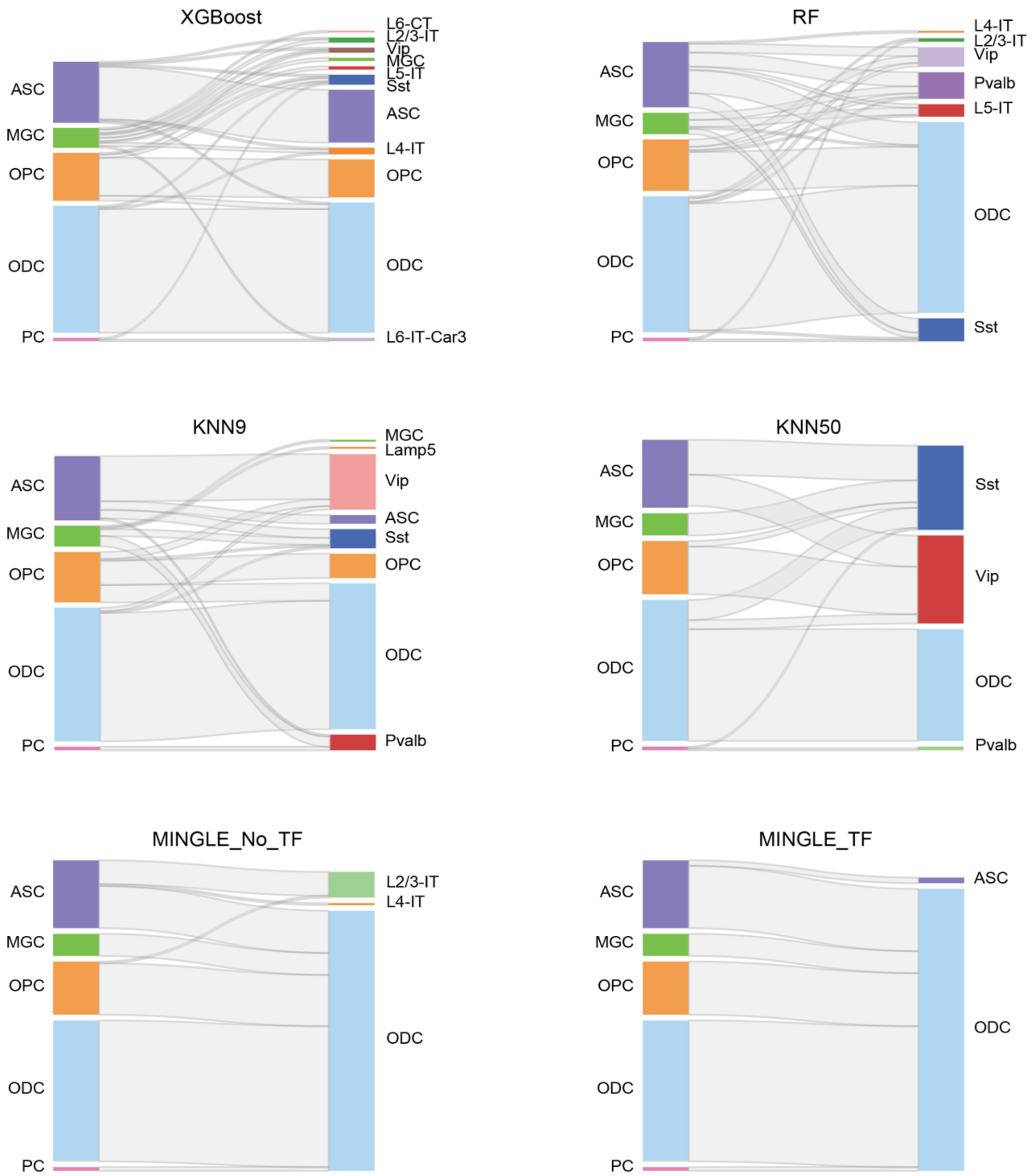

Figure S5. The annotation results of six baseline methods (XGBoost, RF, KNN9, KNN50, MINGLE_No_TF, MINGLE_TF) for non-neuronal cell types in the HB-MTG-2 dataset, presented in Sankey diagrams. Here, all machine learning baselines were implemented with their default parameter configurations.

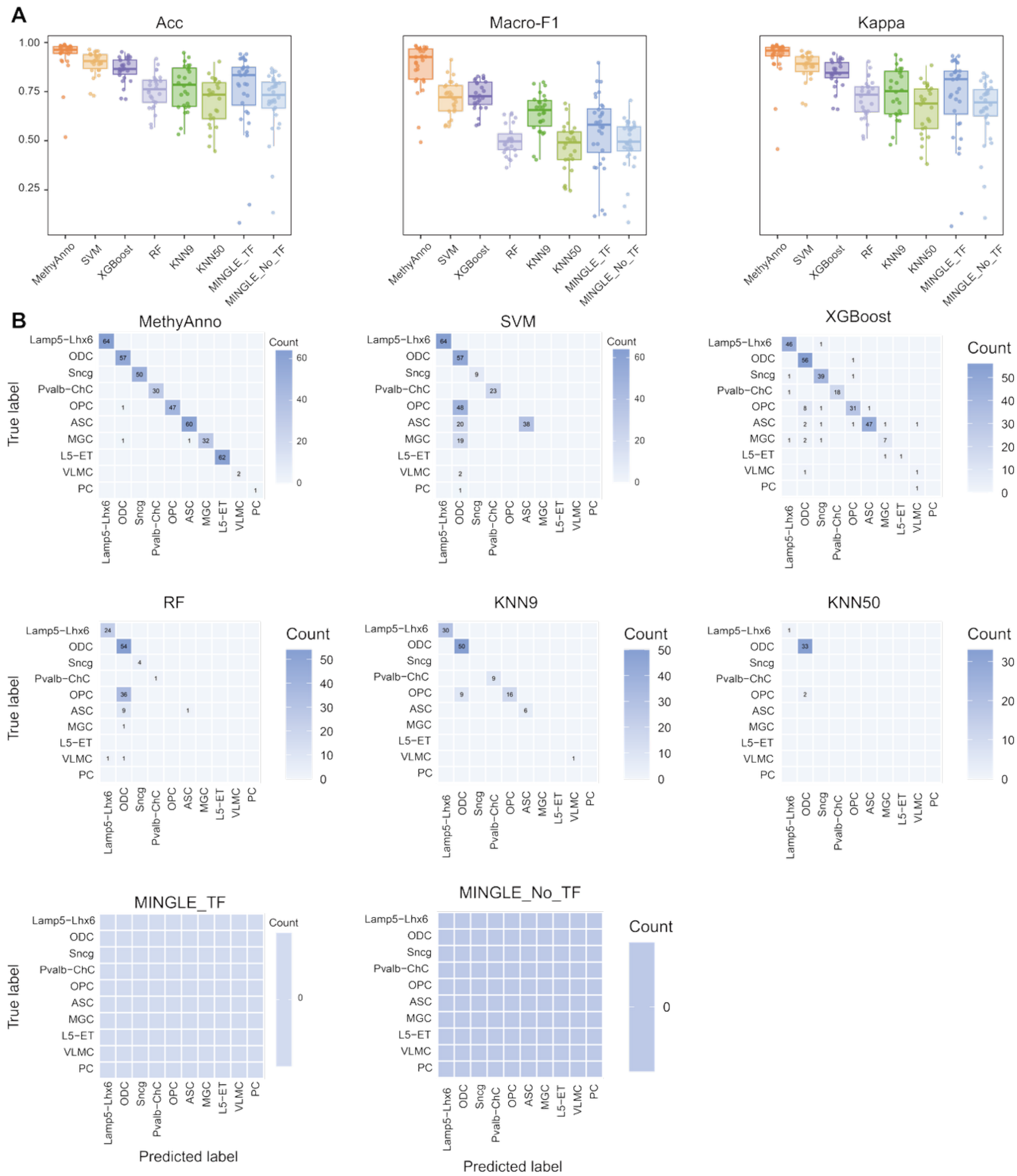

Figure S6. Prediction results of MethyAnno and baseline methods (XGBoost, RF, KNN9, KNN50, MINGLE_No_TF), which were trained on the HB-MTG-1 dataset and tested on the HB-ACC-1 dataset. Here, all machine learning baselines were implemented with their default parameter configurations. Only cell types with fewer than 50 training cells are visualized, where each value represents the number of cells from the column cell type predicted as the corresponding row cell type.

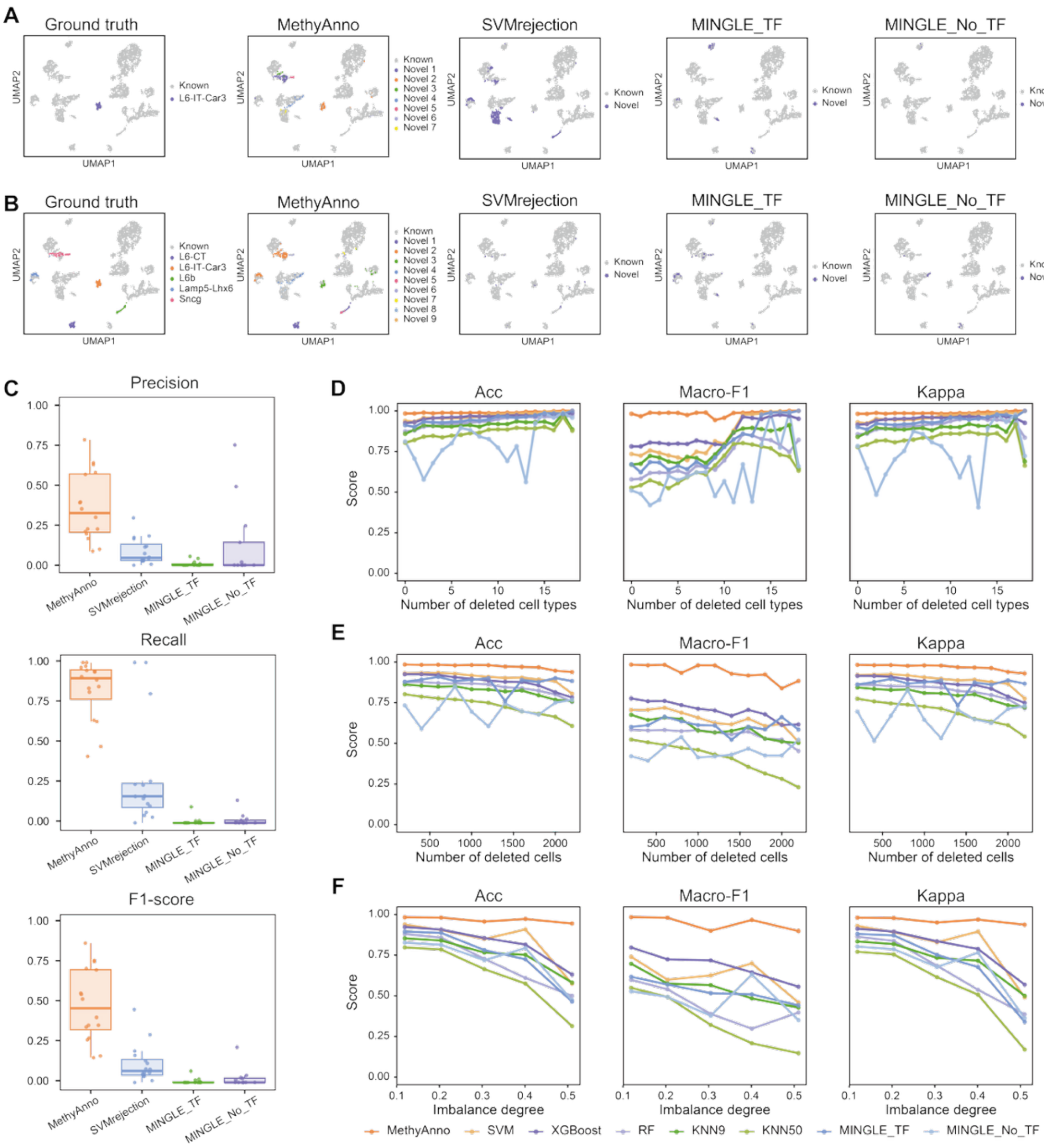

Figure S7. The results of novel type discovery and robustness of MethyAnno, where all machine learning baselines were implemented with their default parameter configurations. **A, B.** UMAP visualizations of the HB-MTG-2 test set (reduced to 50 dimensions via PCA), illustrating the distribution of simulated novel types and the prediction results of MethyAnno and baseline methods. The scenarios include a single novel cell type (A) and five novel cell types (B). **C.** Performance of MethyAnno, SVMrejection and MINGLE in novel cell type identification. Each data point in the box plots represents an experiment where a single specific cell type was excluded from the training set to simulate a novel class. **D–F.** Robustness analysis of MethyAnno and baseline methods (with default parameters) under varying data configurations. The plots display performance trends with respect to the number of deleted cell types (D), the number of deleted cells (E), and the degree of class imbalance (F).

Supplementary Tables

Table S1. Performance comparison of MethyAnno and its ablation variants across three tissues using 10 random seeds.

Tissue	Ablation variant	Acc	Kappa	Macro-F1
Human adipose	MethyAnno	0.9820 ± 0.0012	0.9774 ± 0.0016	0.9522 ± 0.0058
Human adipose	Single branch	0.9806 ± 0.0014	0.9756 ± 0.0017	0.9447 ± 0.0071
Human adipose	No cross-attention	0.9818 ± 0.0019	0.9771 ± 0.0024	0.9456 ± 0.0131
Human adipose	No supervised contrastive loss	0.9806 ± 0.0021	0.9755 ± 0.0027	0.9453 ± 0.0114
Human adipose	No subspace structuring loss	0.9817 ± 0.0020	0.9770 ± 0.0025	0.9494 ± 0.0124
Human adipose	No orthogonality constraint	0.9813 ± 0.0012	0.9765 ± 0.0015	0.9492 ± 0.0070
Human adipose	Single vector	0.9820 ± 0.0023	0.9774 ± 0.0029	0.9531 ± 0.0096
Mouse brain	MethyAnno	0.9633 ± 0.0025	0.9562 ± 0.0030	0.8206 ± 0.0367
Mouse brain	Single branch	0.9584 ± 0.0019	0.9504 ± 0.0022	0.7872 ± 0.0295
Mouse brain	No cross-attention	0.9622 ± 0.0027	0.9550 ± 0.0033	0.8225 ± 0.0507
Mouse brain	No supervised contrastive loss	0.9621 ± 0.0046	0.9547 ± 0.0055	0.8078 ± 0.0560
Mouse brain	No subspace structuring loss	0.9628 ± 0.0018	0.9556 ± 0.0022	0.8124 ± 0.0464
Mouse brain	No orthogonality constraint	0.9635 ± 0.0031	0.9565 ± 0.0037	0.8227 ± 0.0417
Mouse brain	Single vector	0.9638 ± 0.0030	0.9568 ± 0.0035	0.8378 ± 0.0494
Human brain	MethyAnno	0.9849 ± 0.0015	0.9829 ± 0.0016	0.9818 ± 0.0062
Human brain	Single branch	0.9813 ± 0.0015	0.9788 ± 0.0017	0.9346 ± 0.0126
Human brain	No cross-attention	0.9833 ± 0.0017	0.9811 ± 0.0019	0.9726 ± 0.0168
Human brain	No supervised contrastive loss	0.9842 ± 0.0010	0.9821 ± 0.0012	0.9804 ± 0.0078
Human brain	No subspace structuring loss	0.9852 ± 0.0011	0.9832 ± 0.0013	0.9837 ± 0.0023
Human brain	No orthogonality constraint	0.9849 ± 0.0010	0.9829 ± 0.0012	0.9774 ± 0.0158
Human brain	Single vector	0.9843 ± 0.0015	0.9822 ± 0.0017	0.9806 ± 0.0079

Table S2. The summary of hyperparameter configurations used in the MethyAnno framework.

Module	Hyperparameter	Description	Value
Network architecture	D_1	Input dimensionality for the fine-scale methylation features	5000
Network architecture	D_2	Input dimensionality for the coarse-scale methylation features	3000
Network architecture	D_{emb}	Dimensionality of the joint latent embedding space	512
Network architecture	k_{basis}	Number of basis vectors per cell-type-specific subspace	8
Network architecture	$p_{dropout}$	Dropout probability applied across all network layers	0.6
Loss function	λ_1	Weight of the supervised contrastive loss (L_{SCL})	0.8
Loss function	λ_2	Weight of the subspace structuring loss (L_{SUB})	0.1
Loss function	λ_3	Weight of the orthogonality constraint (L_{ORTHO})	0.1
Loss function	τ_1	Temperature scalar for the supervised contrastive loss (L_{SCL})	0.8
Loss function	τ_2	Temperature scalar for the subspace structuring loss (L_{SUB})	0.5
Optimization	lr	Initial learning rate for the Adam optimizer	0.001
Optimization	B	Batch size during the training phase	64
Optimization	T_{total}	Total number of training epochs	70
Optimization	λ_{wd}	Weight decay coefficient for the Adam optimizer	0.0001
Optimization	γ	Multiplicative factor for the ExponentialLR learning rate scheduler	0.95

Table S3. The summary of collected datasets.

Dataset	Species	Tissue	Sequencing technology	Number of cells	Number of cell types	Imbalance degree	Accession number
HB-M1C-1	Homo sapiens	Cerebral cortex	snmC-seq3	2074	22	0.1996	GSE168482
HB-M1C-2	Homo sapiens	Cerebral cortex	snmC-seq3	2951	22	0.2363	GSE168645
HB-M1C-snm3c	Homo sapiens	Cerebral cortex	snm3C-seq	2165	22	0.1845	GSE219113
HB-MTG-1	Homo sapiens	Cerebral cortex	snmC-seq3	2533	22	0.1911	GSE168693
HB-MTG-2	Homo sapiens	Cerebral cortex	snmC-seq3	2496	21	0.1858	GSE168734
HB-MTG-snm3c	Homo sapiens	Cerebral cortex	snm3C-seq	3026	22	0.2263	GSE220451
HB-A46-1	Homo sapiens	Cerebral cortex	snmC-seq3	2518	23	0.2173	GSE167577
HB-A46-2	Homo sapiens	Cerebral cortex	snmC-seq3	2892	22	0.2142	GSE167978
HB-ACC-1	Homo sapiens	Cerebral cortex	snmC-seq3	2698	26	0.1686	GSE168066
HB-ACC-2	Homo sapiens	Cerebral cortex	snmC-seq3	3020	23	0.1586	GSE168169
HB-A5-A7-1	Homo sapiens	Cerebral cortex	snmC-seq3	2616	20	0.2257	GSE179129
HB-A5-A7-2	Homo sapiens	Cerebral cortex	snmC-seq3	2786	20	0.2643	GSE179610
MB-MOs-1	Mus musculus	Isocortex	snmC-seq2	1367	21	0.4168	GSE130553
MB-MOs-2	Mus musculus	Isocortex	snmC-seq2	1101	22	0.3900	GSE131930
MB-MOp-1	Mus musculus	Isocortex	snmC-seq2	1262	21	0.2030	GSE131360
MB-MOp-2	Mus musculus	Isocortex	snmC-seq2	1323	21	0.2338	GSE131430
MB-CP-1	Mus musculus	CNU	snmC-seq2	1106	25	0.4488	GSE131393
MB-CP-2	Mus musculus	CNU	snmC-seq2	1433	23	0.4446	GSE131406
MB-AUD-1	Mus musculus	Isocortex	snmC-seq2	1259	30	0.2962	GSE215213
MB-AUD-2	Mus musculus	Isocortex	snmC-seq2	1229	29	0.3345	GSE215342
MB-VIS-1	Mus musculus	Isocortex	snmC-seq2	1114	25	0.3037	GSE230224
MB-VIS-2	Mus musculus	Isocortex	snmC-seq2	1223	30	0.3242	GSE230226
MB-RSP-1	Mus musculus	Isocortex	snmC-seq2	1105	27	0.3018	GSE213221
Human-Adipose-1	Homo sapiens	Adipose	snm3C-seq3	1106	8	0.2166	GSE297262
Human-Adipose-2	Homo sapiens	Adipose	snm3C-seq3	1094	8	0.1710	GSE297262
Human-Adipose-3	Homo sapiens	Adipose	snm3C-seq3	1098	8	0.1711	GSE297262
Human-Adipose-4	Homo sapiens	Adipose	snm3C-seq3	2237	8	0.1729	GSE297262
Human-Adipose-5	Homo sapiens	Adipose	snm3C-seq3	1117	8	0.1613	GSE297262

Table S4. Hyperparameter search space and final value for conventional machine learning baselines.

Model	Hyperparameter	Description	Search range	Final value
RF	n_estimators	Number of trees	20 to 500, step 20	500
RF	criterion	Split quality criterion	All available criterion functions	entropy
RF	max_depth	Maximum tree depth	None and 20 to 500, step 20	220
SVM	C	Regularization parameter	0.1 to 2.0, step 0.1	1.5
SVM	kernel	Kernel function	All available kernel types	linear
SVM	degree	Degree of polynomial kernel	1 to 10, step 1	None
KNN	n_neighbors	Number of neighbors	5 to 100, step 5	5
KNN	weights	Neighbor weighting function	All available weighting functions	distance
KNN	algorithm	Neighbor search algorithm	All available algorithms	kd_tree
XGBoost	n_estimators	Number of boosting rounds	0 to 480, step 40	480
XGBoost	max_depth	Maximum tree depth	0 to 480, step 40	160
XGBoost	max_leaves	Maximum number of leaves	0 to 480, step 40	40

Table S5. Computational overhead of MethyAnno across different operational stages. The table summarizes the runtime and memory footprint metrics for the data preprocessing, model training, and model inference stages. The results are derived from 25 independent cross-donor annotation experiments. The reported metrics include the mean, maximum, and minimum values for execution time in seconds, peak CPU memory usage in megabytes, and peak GPU memory allocation in megabytes. Workstation with one NVIDIA RTX A6000 GPU, 48 GB VRAM. The number of trainable parameters depends on the number of training cell types C: $7{,}713{,}922 + 4{,}096 \times C$.

Stage	Metric	Mean	Max	Min
Data preprocessing	Time (Seconds)	41.97	265.81	7.62
Data preprocessing	CPU peak RSS (MB)	50806.61	95103.61	21914.18
Data preprocessing	GPU peak allocated (MB)	15.60	16.25	0.00
Training	Time (Seconds)	179.66	359.43	76.55
Training	CPU peak RSS (MB)	15411.33	21687.30	8520.91
Training	GPU peak allocated (MB)	302.27	302.60	301.75
Inference	Time (Seconds)	4.04	11.37	2.18
Inference	CPU peak RSS (MB)	15413.66	21687.30	8520.92
Inference	GPU peak allocated (MB)	237.30	237.74	236.62

Table S6. The summary of cell types mentioned in this study.

Cell type	Cell class	Description
L2/3-IT	Telencephalic excitatory neurons	Intratelencephalic projection excitatory neurons in cortex layer 2/3
L4-IT	Telencephalic excitatory neurons	Intratelencephalic projection excitatory neurons in cortex layer 4
L5-ET	Telencephalic excitatory neurons	Extratelencephalic projection excitatory neurons in cortex layer 5
L5-IT	Telencephalic excitatory neurons	Intratelencephalic projection excitatory neurons in cortex layer 5
L6b	Telencephalic excitatory neurons	Excitatory neurons in cortex layer 6b
L6-CT	Telencephalic excitatory neurons	Corticothalamic projection excitatory neurons in cortex layer 6
L6-IT-Car3	Telencephalic excitatory neurons	Excitatory neurons of both intratelencephalic projection neurons in cortex layer 6 and neurons from Cla
ASC	Non-neuronal Cells	Astrocytes
MGC	Non-neuronal Cells	Macro glia
ODC	Non-neuronal Cells	Oligodendrocytes
OPC	Non-neuronal Cells	Oligodendrocyte progenitor cell
PC	Non-neuronal Cells	Perivascular Cell
VLMC	Non-neuronal Cells	Vascular Leptomeningeal Cell
Lamp5	Inhibitory/non-telencephalic neurons	Cortical inhibitory neurons with gene Lamp5 hypomethylated
Lamp5-Lhx6	Inhibitory/non-telencephalic neurons	Cortical inhibitory neurons with genes Lamp5 and Lhx6 hypomethylated
Pvalb	Inhibitory/non-telencephalic neurons	Cortical inhibitory neurons with genes Pvalb hypomethylated
Pvalb-ChC	Inhibitory/non-telencephalic neurons	Chandelier cells
Sncg	Inhibitory/non-telencephalic neurons	Cortical inhibitory neurons, homologous cell types of which in mouse have genes Sncg hypomethylated
Sst	Inhibitory/non-telencephalic neurons	Cortical inhibitory neurons with genes Sst hypomethylated
Vip	Inhibitory/non-telencephalic neurons	Cortical inhibitory neurons with genes Vip hypomethylated

References

1. Abdelaal, T. *et al.* A comparison of automatic cell identification methods for single-cell RNA sequencing data. *Genome biology* **20**, 194 (2019).
2. Ma, W., Su, K. & Wu, H. Evaluation of some aspects in supervised cell type identification for single-cell RNA-seq: classifier, feature selection, and reference construction. *Genome biology* **22**, 264 (2021).
3. Finucane, H. K. *et al.* Partitioning heritability by functional annotation using genome-wide association summary statistics. *Nature genetics* **47**, 1228–1235 (2015).
4. Slowikowski, K., Hu, X. & Raychaudhuri, S. SNPsea: an algorithm to identify cell types, tissues and pathways affected by risk loci. *Bioinformatics* **30**, 2496–2497 (2014).
5. McLean, C. Y. *et al.* GREAT improves functional interpretation of cis-regulatory regions. *Nature biotechnology* **28**, 495–501 (2010).
